# Norbornadiene-functionalized triazatriangulenium and trioxatriangulenium platforms

**DOI:** 10.3762/bjoc.15.175

**Published:** 2019-07-30

**Authors:** Roland Löw, Talina Rusch, Tobias Moje, Fynn Röhricht, Olaf M Magnussen, Rainer Herges

**Affiliations:** 1 Otto Diels Institute for Organic Chemistry, University of Kiel, Otto-Hahn-Platz 4, 24118 Kiel, Germany; 2 Institute for Experimental and Applied Physics, University of Kiel, Leibnizstraße 19, 24098 Kiel, Germany

**Keywords:** [2 + 2] cycloaddition, [2 + 2] cycloreversion, norbornadiene, photochemical isomerization, quadricyclane, self-assembled monolayers, TATA platform, thermal isomerization, TOTA platform

## Abstract

Triazatriangulenium (TATA) and trioxatriangulenium (TOTA) ions are particularly suited systems to mount functional molecules onto atomically flat surfaces such as Au(111). The TATA and TOTA units serve as platforms that absorb onto the surface and form ordered monolayers, while the functional groups are protruding upright and freestanding from the central carbon atoms. Azobenzene derivatized TATA’s are known to exhibit extremely fast *cis*→*trans* isomerization on metal surfaces, via a peculiar non-adiabatic singlet→triplet→singlet mechanism. We now prepared norbornadienes (NBD) and quadricyclanes (QC) attached to TATA and TOTA platforms which can be used to check if these accelerated rates and the spin change mechanism also apply to [2 + 2] cycloreversions (QC→NBD).

## Introduction

Recently, we discovered that the thermochemically forbidden *cis*–*trans* isomerization of azobenzenes can be efficiently catalysed by a very peculiar mechanism on bulk gold [1]. In heterogeneous catalysis, the surface is chemically involved in bond making and breaking. In contrast to this conventional surface catalysis the new mechanism does not involve direct contact with the surface. Electronic coupling via a conjugated π-system to the conducting band of a bulk metal is sufficient to accelerate the rate of isomerization by three orders of magnitude [2–4]. To keep the reacting azobenzene molecule at a distance of about 14 Å from the surface, it is not directly absorbed onto the surface but mounted on a carefully designed molecular framework. This approach was named the platform approach [5]. Three molecular units are combined in a modular way to achieve a controlled adsorption on the surface: the platform, a spacer and the functional molecule. Triazatriangulenium (TATA) and trioxatriangulenium (TOTA) units are used as molecular platforms. They adsorb on the surface and form ordered monolayers. A linear, π-conjugated spacer (e.g., an ethynyl unit) is attached to the central carbon atom and the functional molecule is mounted on top. This architecture allows investigating molecules on surfaces under controlled conditions. The size of the platforms determines the intermolecular distances and enforces an upright orientation of the free standing functional groups [6]. The length and the nature of the spacer is used to tune the distance and electronic coupling of the functional system on top [1].

Preliminary experiments proved that the electronic coupling to the surface is the decisive parameter controlling the *cis–trans* rate acceleration of azobenzenes and not the length of the spacer [1]. A full conjugation path from the azobenzene on top through the ethynyl spacer and the platform to a bulk gold surface shortens the half-life of the metastable *cis-*isomer from days to seconds even though the azo N=N group is 11 bonds and 14 Å away from the surface. A singlet→triplet→singlet pathway was suggested to explain the dramatic rate acceleration.

To obtain further insight into this unusual mechanism and to explore the scope and limitation of the general concept, we are aiming at the extension from simple *cis*→*trans* isomerization to other thermochemically forbidden reactions such as [2 + 2] cycloreversions. Moreover, a deeper understanding of the non-adiabatic, catalytic process and successful application of the concept to the QC→NBD isomerization could contribute to the elucidation of the mechanisms of bulk metal catalysis and open new ways to design new catalytic systems.

**Figure 1:**
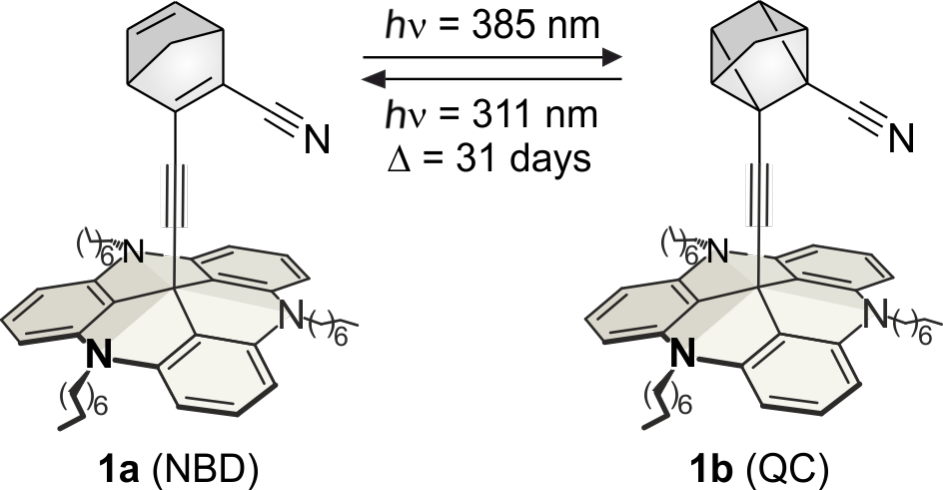
Structures of the norbornadiene platform **1a** and the quadricyclane platform **1b** (for geometry coordinates and further details see Supporting Information File 1). The norbornadiene isomerizes upon irradiation with 385 nm to the quadricyclane **1b**. Back-isomerization to the norbornadiene **1a** is achieved upon irradiation with 311 nm or thermochemically with a half-life of 31 days.

Towards this end, and following the “platform concept”, we designed a cyano-substituted norbornadiene, which is functionalized with an acetylene spacer on a TATA platform to investigate an eventual “spin-catalysed” [2 + 2] cycloreversion on bulk gold of quadricyclane **1b** to norbornadiene **1a** (Figure 1).

The cycloreversion of most quadricyclane systems proceeds smoothly in solution upon irradiation in the presence of triplet sensitizers [7]. If **1b** is adsorbed on a gold surface the bulk gold could take the role of a triplet sensitizer, mediate the spin change (which otherwise is forbidden) and accelerate the cycloreversion. Substitution in the 2 and 3 positions shifts the bathochromic absorption to 375 nm which is in agreement with similar systems [8]. Furthermore, the cyano and ethynyl groups provide a complete conjugation path across the double bond of norbornadiene to the metal. Additionally, it is known that electron-withdrawing groups in 2 or 3 position change the triplet energy hypersurface in such a way that a triplet excited quadricyclane **1b** decays into the ground state of the norbornadiene **1a** [9], which is a precondition for an efficient QC→NBD isomerization via our postulated non-adiabatic singlet→triplet→singlet mechanism.

## Results and Discussion

If our proposed spin change mechanism, mediated by the conducting electrons in bulk gold were correct, interruption of the conjugation path and decoupling from the surface should restore the properties of the system (half-life of the metastable quadricyclane) in solution. According to this line of thought, we synthesized molecule **2** with a 2-methylphenyl group inserted into the spacer unit (see below Scheme 2). The methyl group prevents a planar arrangement of the phenyl group and the double bond of the NBD unit and thus lowers the conjugation.

We also synthesized the corresponding system directly connected to a TOTA platform **3**. Functionalized TOTA molecules are more stable than the corresponding TATA systems and usually can be sublimed without decomposition, which is a necessary precondition for ultra-high vacuum STM investigations.

**Scheme 1:**
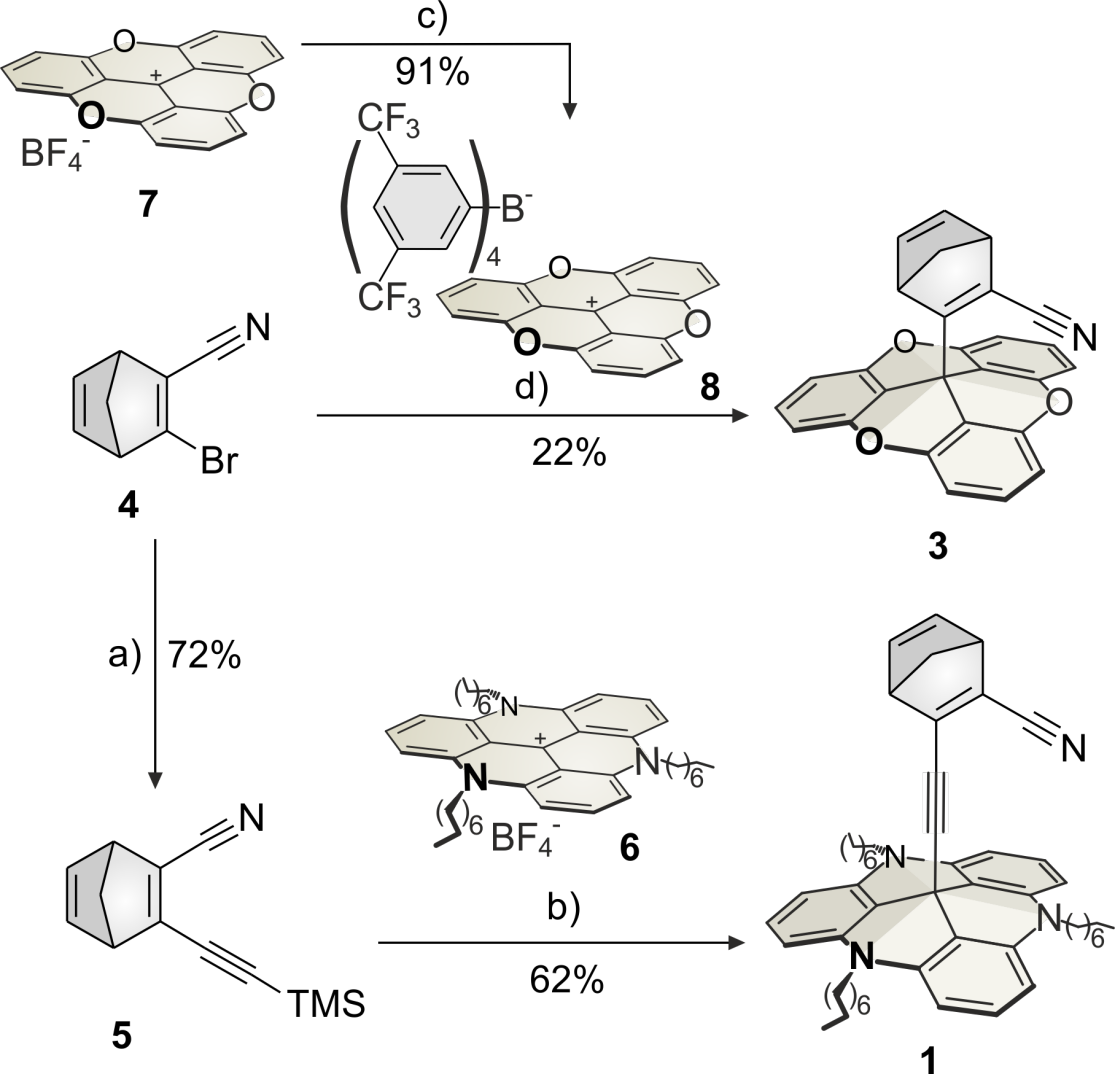
Syntheses of the norbornadiene TATA platform **1** and TOTA platform **3**. a) TMS-acetylene, Pd(PPh_3_)_4_, Cu(I)I, Et_3_N, toluene, N_2_, 60 °C, 1.3 h; b) KOH, THF, N_2_, reflux, 5 h; c) sodium tetrakis[3,5-bis(trifluoromethyl)phenyl]borate (NaBAr^F^_4_), DCM, rt, 2 h; d) *n*-BuLi, THF, N_2_, −78 °C to rt, 20 h.

The 3-bromo-2-cyano-substituted norbornadiene **4** was synthesized as described in the literature (Scheme 1) [10–12].

**4** was converted to **5** with trimethylsilylacetylene (72%) in a Sonogashira cross-coupling reaction. The triazatriangulenium ion **6** was synthesized according to a procedure of Laursen and Krebs [13]. The platform **6** was functionalized with norbornadiene **5** by deprotection of the acetylene with potassium hydroxide and in situ formation of the C–C bond between the acetylene and the central C atom of the platform **6** to yield the norbornadiene-substituted platform **1** (62%).

**Scheme 2:**
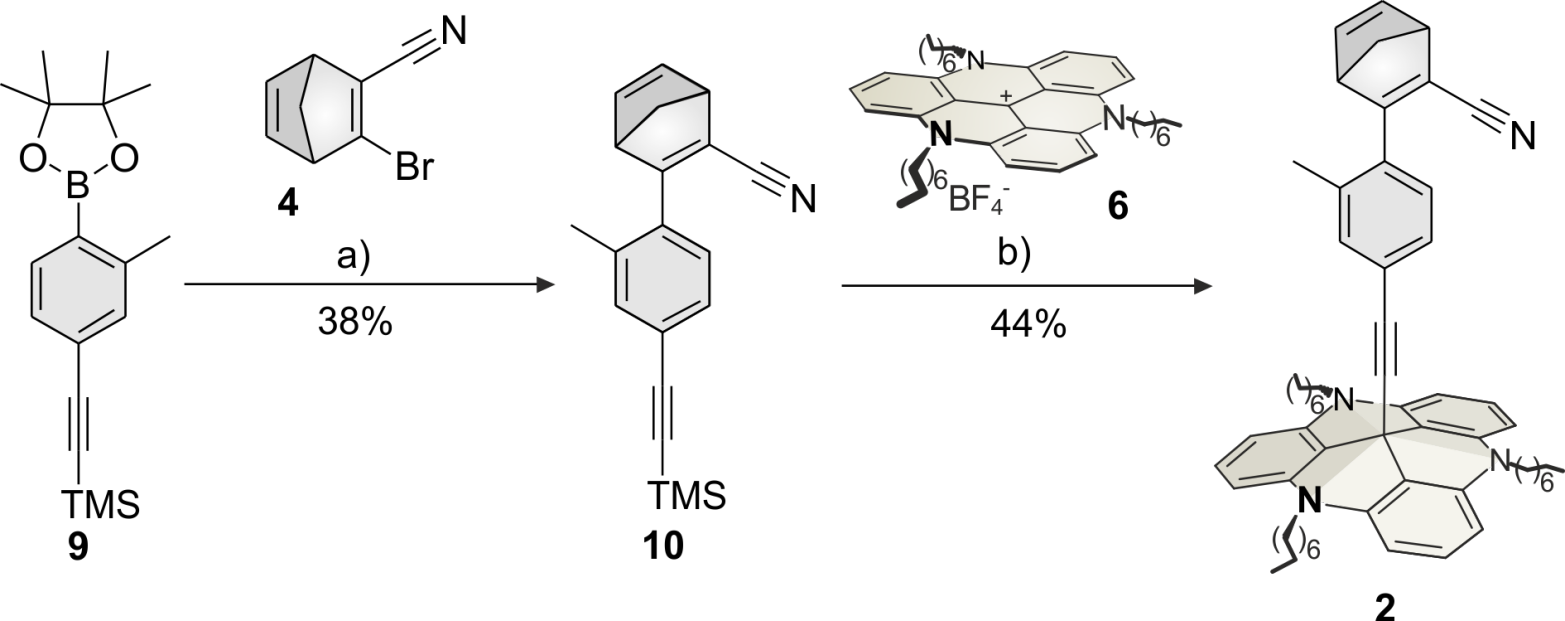
Synthesis of methylphenylnorbornadiene platform **2**. a) Pd(PPh_3_)_4_, Na_2_CO_3_, toluene, EtOH, H_2_O, N_2_, reflux, 12 h; b) KOH, THF, N_2_, reflux, 1 h.

The TOTA cation with the tetrakis(3,5-bis(trifluoromethyl)phenyl)borate anion (TOTA^+^ [BAr^F^_4_]^−^, **8**) was obtained by ion exchange of the TOTA tetrafluoroborate **7** (TOTA^+^ BF_4_^−^) to achieve a high solubility in organic solvents [14]. 3-Bromo-2-cyanonorbornadiene (**4**) was subjected to halogen–metal exchange with *n*-BuLi and coupled with the central atom of the TOTA platform **8** to obtain the norbornadiene-functionalized TOTA platform **3** (22%, Scheme 1). The synthesis of the corresponding TATA platform including an additional 2-methylphenyl group (**2**) was obtained in a convergent synthesis (Scheme 2). Boronic ester **9** was synthesized as described in the literature [15]. In a Suzuki cross-coupling reaction norbornadiene **4** was coupled with **9** to the extended norbornadiene **10** (38%), which was attached to the TATA platform **6** to yield the extended norbornadiene platform **2** (44%).

**Figure 2:**
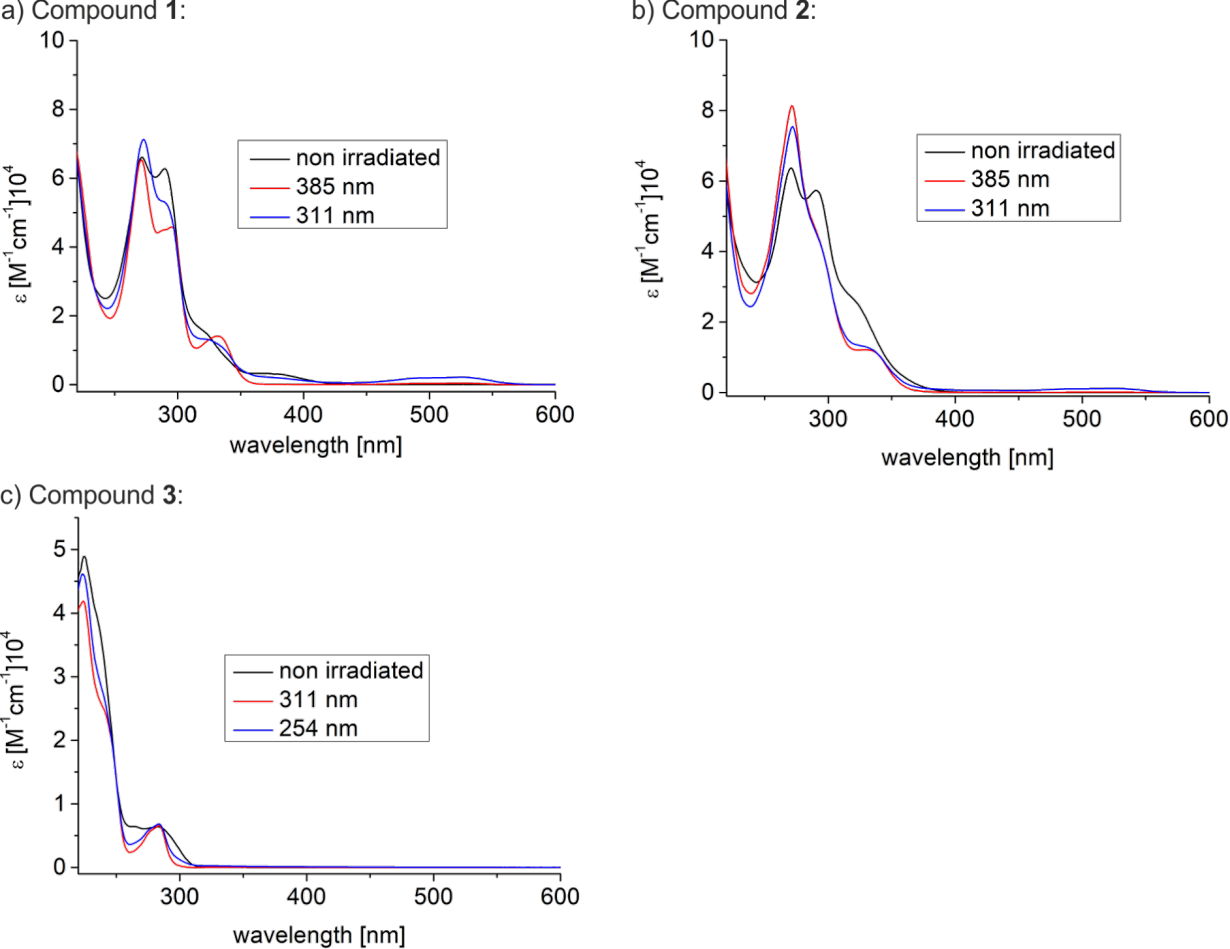
UV–vis spectra of platform molecules **1** (a), **2** (b) and **3** (c) (in THF at rt): Norbornadiene (black), quadricyclane (red) and after irradiation with 311 nm (compound **1b** and **2b**) or 254 nm (compound **3b**) for 2.5 min (blue).

The photophysical properties and the switching behaviour of **1**, **2** and **3** were determined in solution (THF). The UV–vis spectra of the norbornadienes (NBD, black, **1a**, **2a**, **3a**) and quadricylanes (QC, red, **1b**, **2b**, **3b**) and the spectra of the QCs after irradiation with 311 nm for **1b** and **2b** or 254 nm for **3b** (blue) are shown in Figure 2. The bathochromic absorption maximum of norbornadiene **1a** is at 375 nm (as compared to <300 nm in parent norbornadiene) [16–17]. The absorption maximum of quadricyclane **1b** is at 336 nm (as compared to 187 nm in parent quadricyclane) [18]. The weak and broad absorption band with a maximum at 524 nm is due to the TATA cation generated by decomposition during irradiation with 311 nm.

**Figure 3:**
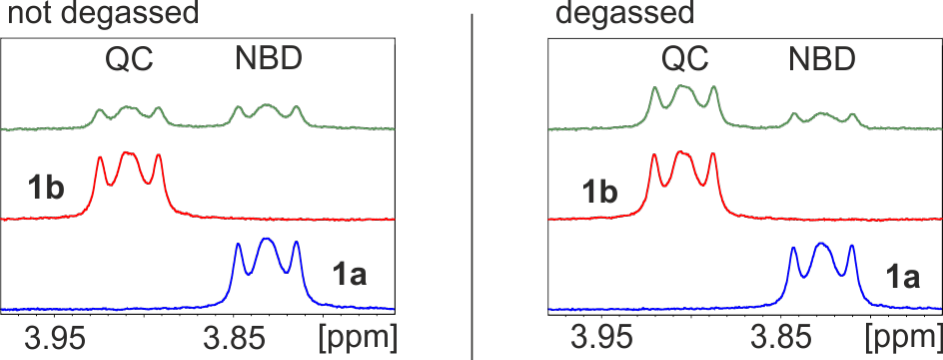
^1^H NMR spectra of **1** in deuterated oxygen containing deuterated benzene (left) and degassed deuterated benzene (right). Shown are the signals of the CH_2_ groups of the alkyl side chains next to the nitrogen atoms in the TATA platforms, which are indicative of the isomerization. Left bottom (blue): non-irradiated NBD **1a**; left middle (red): pure QC **1b** obtained after irradiation of **1b** with 385 nm for one minute; and left top (green): photostationary state of **1a** and **1b** after 1 h irradiation with 311 nm under air (52% **1a**/48% **1b**). Right bottom (blue): pure NBD **1a**; right middle (red): pure QC **1b** obtained after irradiation of **1a** with 385 nm for one minute and right top (green): photostationary state of **1a** and **1b** after 2 h irradiation with 311 nm under nitrogen atmosphere (28% **1a**/72% **1b**).

The photostationary states were determined in deuterated oxygen containing deuterated benzene and degassed deuterated benzene by ^1^H NMR measurements (Figure 3). Norbornadiene **1a** isomerizes quantitatively to quadricyclane **1b** by irradiation with 385 nm. Upon irradiation of **1b** under nitrogen with 311 nm, the cycloreversion yields 28% norbornadiene (Table 1).

The efficiency of the cycloreversion is higher under air (52%), however, slow decomposition was observed (cleavage of the TATA^+^ platform). Obviously, in the presence of oxygen, the photochemical cycloreversion proceeds via a triplet radical mechanism. This agrees with observations described in the literature [16]. In degassed benzene neither **1a** nor **1b** exhibits decomposition upon repeated irradiation with 385 nm and 311 nm.

**Figure 4:**
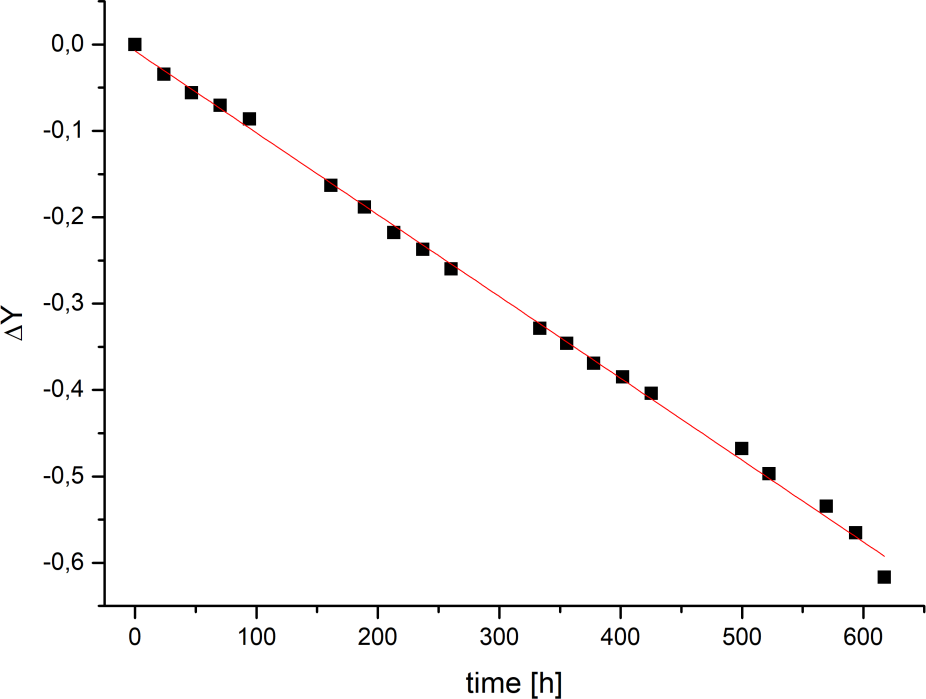
Determination of the thermal isomerization rate *k* of **1b** (QC) by ^1^H NMR spectroscopy (toluene, 293.5 K, 800 μmol/L, under nitrogen). ΔY: ln {[QC]_t_/[QC]_0_}, [QC]_t_: ^1^H NMR integral of the CH_2_ group neighbouring the N bridge atom of the TATA platform in QC **1b** at time *t*, [QC]_0_ corresponding ^1^H integral at *t* = 0. A rate constant of *k* = 0.95·10^−3^ s^−1^ was determined from a linear fit of the ΔY/*t* curve.

The thermal isomerization of QC **1b** back to NBD **1a** was investigated by ^1^H NMR measurements (Figure 4).

The cycloreversion follows a first order reaction, the rate constant could be determined by logarithmic fitting of the integrals of the CH_2_ signals next to the nitrogen atoms of the platform as *k* = 1.06·10^−3^ s^−1^ under air and 0.95·10^−3^ s^−1^ under nitrogen. Hence, the thermal reaction (in contrast to the photochemical reaction) is not largely affected by oxygen. The half-life of the metastable quadricyclane is *t*_1/2_ = 655 h in benzene under air at 293 K (Table 1). Minor amounts of degradation products (<1%) after following the cycloreversion within a period of one month are visible in the ^1^H NMR spectrum. Under nitrogen atmosphere the half-life of the cycloreversion is *t*_1/2_ = 732 h (294 K).

**Table 1:** Photostationary states (PSS) of norbornadiene platforms **1**, **2** and **3** upon irradiation with light of the wavelengths **λ**_irrad_ = 385 nm (**1a**, **2a**) or 311 nm (**3a**) and 311 nm (**1b**, **2b**) or 254 nm (**3b**) and the thermal isomerization half-life *t*_1/2_ determined by ^1^H NMR spectroscopy (deuterated benzene under air/degassed deuterated benzene).

Compound	atmosphere	PSS % QC (λ_irrad_)	PSS % NBD (λ_irrad_)	*t*_1/2_ (h)	EA kJ/mol

**1**	air	≈100 (385 nm)	52 (311 nm)	655 (293 K)	/
**1**	N_2_	≈100 (385 nm)	28 (311 nm)	732 (294 K)	111
**2**	N_2_	≈100 (385 nm)	48 (311 nm)	>1 year	/
**3**	N_2_	91 (311 nm)	33 (254 nm)	>1 year	/

The rate constant as a function of the temperature follows an Arrhenius-type relationship. The activation energy for the cycloreversion was determined by linear fit of ln (*k*) as a function of 1/*T*. The cycloreversion of QC **1b** to NBD **1a** has an activation energy of 111 kJ mol^−1^ (degassed deuterated benzene). The switching efficiency of NBD **2a** to QC **2b** is quantitative (≈100%) after irradiation with 385 nm, whereas the photostationary state of NBD **3a** to QC **3b** is lower with 91%.

No thermal cycloreversion of the quadricyclanes **2b** and **3b** was observed at room temperature within a period of one month. Obviously, a cyano as well as a neighbouring ethynyl substituent are necessary to induce the back-isomerisation at ambient conditions as realized in compound **1**. Consequently, future surface chemistry investigations will be performed with compound **1** including the TATA platform and an ethynyl spacer.

## STM Measurements

**Figure 5:**
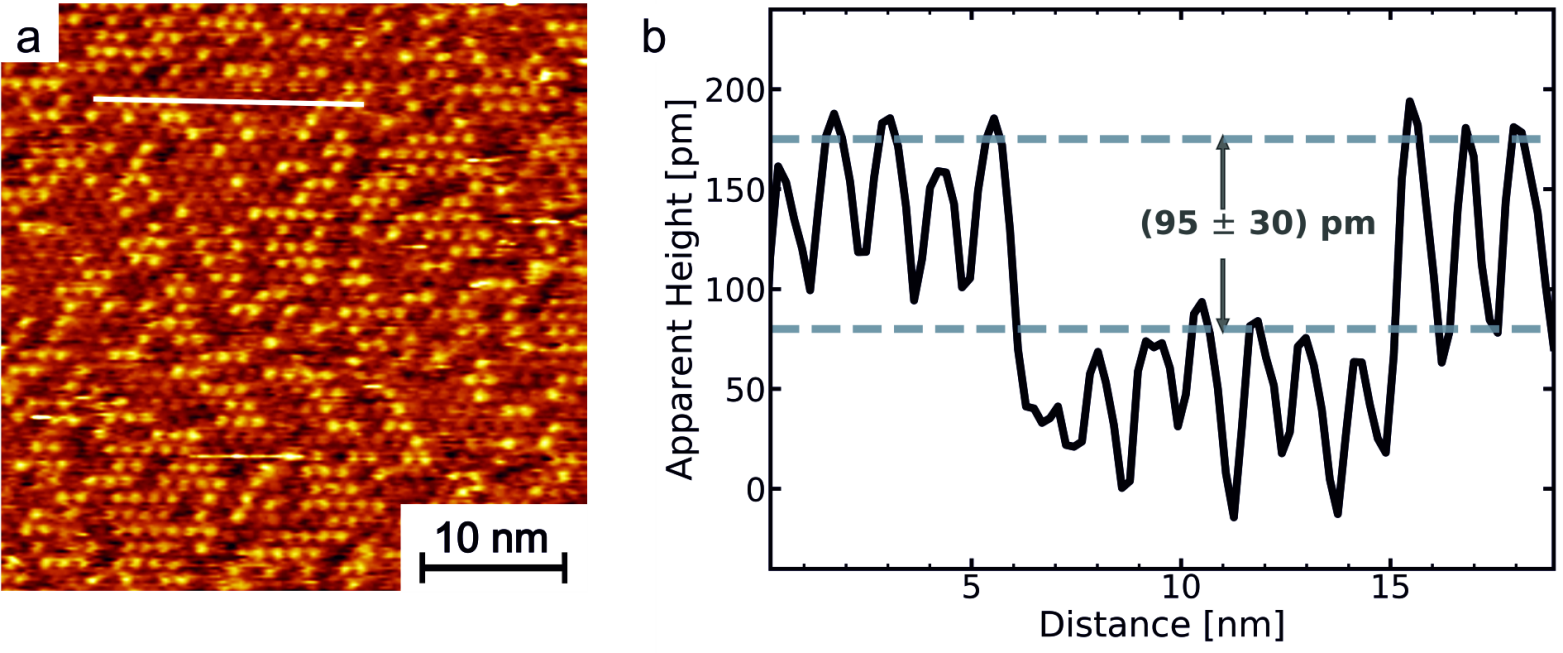
(a) STM image of self-assembled monolayers of compound **1** on Au(111) (40 × 40 nm^2^, *I*_t_ = 0.05 nA, *U*_bias_ = 0.40 V) and (b) crossection along the white line in (a).

The adsorption behaviour of the NBD **1a** on Au(111) surfaces was studied by scanning tunnelling microscopy (STM) at room temperature (Figure 5a). The molecules form a hexagonally ordered self-assembled monolayer (SAM) with an intermolecular distance of (1.23 ± 0.07) nm. This is in agreement with a (√19 × √19) R23.4° superstructure, which was observed in our previous investigations of adlayers of octyl-TATA derivatives [1,6,19–21]. Two types of molecules with a distinct difference in apparent height of ≈1 Å were observed in the STM images (Figure 5b). Both types of molecules are located at identical positions of the (√19 × √19) R23.4° lattice and seem to be distributed rather randomly on the surface. Two explanations are possible to account for the presence of these two species: Either they correspond to a mixture of adsorbed NBD-TATA **1a** and QC-TATAs **1b** or to coadsorption of **1a** and the pure octyl-TATA **6**. The first case may be possible, because the molecules are able to switch to the **1b** state at a wavelength of 385 nm and the substance was exposed to daylight during preparation and incorporation into the STM. However, since the ratio on the surface is 42% for molecules with a greater apparent height and 58% for molecules with a lower apparent height, this assumption is unlikely as at least 42% of the molecules would have to be in the switched state **1b**. The second explanation, i.e., that the mixed monolayer consists of octyl-TATA **6** and the NBD-TATA **1a**, seems to be more likely, since molecule **1** decays slowly under irradiation in contact with oxygen. Previous measurements have shown that self-assembly from solutions containing pure and vertically functionalized TATA molecules leads to the formation of stochastically mixed monolayers with a highly ordered (√19 × √19) R23.4° superstructure [22]. This would be also expected for self-assembly from solution containing **1a** and **6**. The composition of the adlayer on the surface does not necessarily have to be identical to the ratio of the two species in solution. In fact, in our previous study the fraction of adsorbed octyl-TATA **6** as compared to that of the functionalized TATA was found to be much higher than the ratio in solution. We attributed this to the smaller size of **6** and its correspondingly higher diffusion coefficient, which results in faster transport to the Au surface and accordingly enhanced surface coverages. Thus, even low decay rates of **1a** may lead to sufficiently high concentrations of **6** for obtaining mixed adlayers. By varying the preparation conditions, e.g., performing the preparation in a nitrogen atmosphere, decomposition might be avoided and monolayers of better quality could be achievable.

## Conclusion

In summary, we present the syntheses of three different norbornadiene functionalized platform molecules **1**–**3**. The photochemical switching between the norbornadiene and quadricyclane isomers with two different wavelengths was investigated. Norbornadienes **1** and **2** are quantitatively converted to the corresponding quadricyclanes upon irradiation with light of 385 nm. Back-isomerization with 311 nm to the norbornadiene isomer **1a** is less efficient (52%). The high-energy quadricyclane isomer **1b** is thermochemically unstable at room temperature (half-life 31 d at 21 °C in benzene) only if a cyano as well as an ethynyl substituent are present. No thermal conversion under ambient conditions was observed for **2** and **3**, which are lacking ethynyl substitution. Further studies will be devoted to the surface chemistry of these compounds. Norbornadiene **1** forms highly ordered monolayers on Au(111) surfaces with two different apparent heights. It is not clear if a mixture of norbornadiene **1a** and quadricyclane **1b** or norbornadiene **1a** and octyl-TATA **6** form these mixed monolayers.

## Experimental

For detailed experimental procedures, including NMR, UV–vis, MS spectra and STM measurements see Supporting Information File 1, chapters I–IV, and for kinetic studies see chapter V.

## Supporting Information

File 1 Experimental and analytical data.

## References

[1] Schlimm A, Löw R, Rusch T, Röhricht F, Strunskus T, Tellkamp T, Sönnichsen F, Manthe U, Magnussen O, Tuczek F (2019). Angew Chem, Int Ed.

[2] Jung U, Schütt C, Filinova O, Kubitschke J, Herges R, Magnussen O (2012). J Phys Chem C.

[3] Jacob H, Ulrich S, Jung U, Lemke S, Rusch T, Schütt C, Petersen F, Strunskus T, Magnussen O, Herges R (2014). Phys Chem Chem Phys.

[4] Bronner C, Tegeder P (2014). New J Phys.

[5] Baisch B, Raffa D, Jung U, Magnussen O M, Nicolas C, Lacour J, Kubitschke J, Herges R (2009). J Am Chem Soc.

[6] Ulrich S, Jung U, Strunskus T, Schütt C, Bloedorn A, Lemke S, Ludwig E, Kipp L, Faupel F, Magnussen O (2015). Phys Chem Chem Phys.

[7] Nishino H, Toki S, Takamuku S (1986). J Am Chem Soc.

[8] Quant M, Lennartson A, Dreos A, Kuisma M, Erhart P, Börjesson K, Moth-Poulsen K (2016). Chem – Eur J.

[9] Ikezawa H, Kutal C, Yasufuku K, Yamazaki H (1986). J Am Chem Soc.

[10] Kenndoff J, Polborn K, Szeimies G (1990). J Am Chem Soc.

[11] Tranmer G K, Yip C, Handerson S, Jordan R W, Tam W (2000). Can J Chem.

[12] Gunes Y, Arcelik N, Sahin E, Fleming F F, Altundas R (2015). Eur J Org Chem.

[13] Laursen B W, Krebs F C (2001). Chem – Eur J.

[14] Martin J C, Smith R G (1964). J Am Chem Soc.

[15] Browne D L, Baumann M, Harji B H, Baxendale I R, Ley S V (2011). Org Lett.

[16] Gray V, Lennartson A, Ratanalert P, Börjesson K, Moth-Poulsen K (2014). Chem Commun.

[17] Dilling W L (1966). Chem Rev.

[18] Srinivasan R, Baum T, Epling G (1982). J Chem Soc, Chem Commun.

[19] Hammerich M, Rusch T, Krekiehn N R, Bloedorn A, Magnussen O M, Herges R (2016). ChemPhysChem.

[20] Schlimm A, Stucke N, Flöser B M, Rusch T, Krahmer J, Näther C, Strunskus T, Magnussen O M, Tuczek F (2018). Chem – Eur J.

[21] Lemke S, Ulrich S, Claußen F, Bloedorn A, Jung U, Herges R, Magnussen O M (2015). Surf Sci.

[22] Rusch T R, Hammerich M, Herges R, Magnussen O M

