# Supplementary material for: Norbornadiene-functionalized triazatriangulenium and trioxatriangulenium platforms
Source: Beilstein J Org Chem. 2019 Jul 30;15:1815–21. doi: 10.3762/bjoc.15.175 (PMC6693376; doi:10.3762/bjoc.15.175)
Supplement: File 1 — Experimental and analytical data. [file Beilstein_J_Org_Chem-15-1815-s001.pdf]

BEILSTEIN JOURNAL OF ORGANIC CHEMISTRY

Supporting Information

for

Norbornadiene-functionalized triazatriangulenium and trioxatriangulenium platforms

Roland Löw, Talina Rusch, Tobias Moje, Fynn Röhricht, Olaf M. Magnussen and Rainer Herges

Beilstein J. Org. Chem. **2019,** *15,* 1815–1821. doi:10.3762/bjoc.15.175

Experimental and analytical data

License and Terms: This is a supporting information file under the terms of the Creative Commons Attribution License (http://creativecommons.org/licenses/by/4.0). Please note that the reuse, redistribution and reproduction in particular requires that the authors and source are credited.
The license is subject to the *Beilstein Journal of Organic Chemistry* terms and conditions: (https://www.beilstein-journals.org/bjoc)

Table of Contents

I. Analytical equipment and methods

II. Experimental procedures

III. NMR spectra

IV. UV–vis absorption spectra

V. Kinetic studies in solution by ^{1}H NMR spectroscopy

VI. STM measurements

VII. Calculations

I. Analytical equipment and methods

Materials

Solvents for extraction and chromatography were technical grade. Most solvents used in reactions were extra dried (abs.) or used as received. Analytical TLC was performed with Polygeram® SilG/UV254 (Macherey Nagel, 0.2 mm particle size) and visualization was accomplished by UV light. Flash chromatography was carried out using 0.040–0.063 nm silica gel (Merck). Reactions were carried out inert atmosphere using nitrogen (N_2) as gas.

NMR Spectroscopy

NMR spectra were measured in deuterated solvents (Deutero). All compounds were characterized using ^{1}H and ^{13}C NMR spectroscopy. The signals were assigned using 2D spectroscopy. For ^{1}H and ^{13}C NMR assignment we performed HSQC and HMBC experiments. The degree of deuteration is given in parentheses. ^{1}H NMR spectra are referenced to the following signals:

chloroform-*d* (99.8%): δ = 7.26 ppm. (s)

benzene-d_6 (99.8%): δ = 7.16 ppm. (s)

acetone-d_6 (99.5%): δ = 2.05 ppm. (quint.)

The signal multiplicities are abbreviated as follows:

s: singlet, d: doublet, t: triplet, m: multiplet, dt: double triplet, ps. t: pseudo triplet, dd: double dublet, td: trible dublet.

Measurements were performed by the following instruments:

Bruker CABAV 500neo (^{1}H NMR: 500 MHz, ^{13}C NMR: 125 MHz, ^{11}B NMR: 160 MHz, ^{19}F NMR: 470 MHz, ^{29}Si NMR: 99 MHz)

Bruker AV 600 (^{1}H NMR: 600 MHz, ^{13}C NMR: 150 MHz)

IR spectroscopy

Infrared spectra were measured on a Perkin-Elmer 1600 Series FT-IR spectrometer with an A531-G Golden-Gate-Diamond-ATR-unit. Signals were abbreviated with w, m, s and for weak, medium and strong intensities. Broad signals are additionally labeled with br.

Mass spectrometry

The high resolution (HR) mass spectra were measured with an APEX 3 FT-ICR with a 7.05 T magnet by co. Bruker Daltonics. Electron impact (EI). Electrospray ionization (ESI) mass spectra were measured with a Thermo Scientific Q EXACTIVE.

Chromatrography stationary phases

For column chromatography purifications silica gel (Merck, particle size 0.040–0.063 mm) was used. R_f values were determined by thin layer chromatography onPolygram® Sil G/UV254 (Macherey-Nagel, 0.2 mm particle size).

II. Experimental procedures

II.1 3-[2-(Trimethylsilyl)ethynyl]bicyclo[2.2.1]hepta-2,5-diene-2-carbonitrile (5).

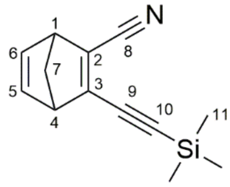

In toluene (24 mL), 3-bromobicyclo[2.2.1]hepta-2,5-diene-2-carbonitrile (**4** [1], 600 mg, 3.06 mmol) was dissolved under nitrogen atmosphere, trimethylsilylacetylene (522 µL, 3.67 mmol), $Pd(PPh_3)_4$ (106 mg, 91.8 µmol), copper(I) iodide (58.3 mg, 306 µmol) and triethylamine (1.06 mL, 7.65 mmol) were added and the mixture was stirred for 80 min at 60 °C. The mixture was filtered through celite and the solvent was removed under reduced pressure. The crude product was purified via column chromatography (silica gel, cyclohexane/ ethyl acetate, 4:1) to obtain a yellow liquid (468 mg, 2.19 mmol, 72%).

^{1}H NMR (500.1 MHz, $CDCl_3$, 298 K, TMS): δ = 6.85-6.81 (m, 2H, *H*-5, *H*-6), 3.86-3.83 (m, 1H, *H*-1), 3.77-3.73 (m, 1H, *H*-4), 2.27 (dt, ^3J = 7.0 Hz, ^4J = 1.6 Hz, 1H, *H*-7$_a$), 2.18 (dt, ^3J = 7.0 Hz, ^4J = 1.6 Hz, 1H, *H*-7$_b$), 0.24 (s, 9H, *H*-11) ppm.

^{13}C NMR (125.8 MHz, $CDCl_3$, 298 K, $CHCl_3$): δ = 154.1 (s, *C*-2), 142.0 (s, *C*-5), 141.5 (s, *C*-6), 129.8 (s, *C*-3), 115.0 (s, *C*-9), 97.6 (s, *C*-8), 73.1 (s, *C*-7), 57.3 (s, *C*-4), 54.2 (s, *C*-1), -0.2 (s, *C*-11) ppm.

^{29}Si NMR (99.4 MHz, $CDCl_3$, 298 K, TMS): δ = -16.23 ppm.

MS (EI, 70eV): m/z = 213.1 $[M]^+$.

IR (ATR)**:** $\tilde{\nu}$ = 2927 (w), 2852 (w), 2207 (m), 2139 (w), 1576 (w), 1557 (w), 1450 (w), 1302 (m), 1251 (m), 1132 (w), 1068 (w), 1019 (w), 840 (vs), 760 (m), 733 (s), 702 (w), 626 (m), 534 (m) cm^{-1}.

HRMS (EI, 70 eV): m/z $[M]^+$ calcd. for $C_{13}H_{15}NSi$: 213.09738, found: 213.09724.

II.2 12c-(2-(2-Cyanobicyclo[2.2.1]hepta-2,5-diene-3-yl)ethynyl)-4,8,12-tri-*n*-octyl-4,8,12-triazatriangulene (1).

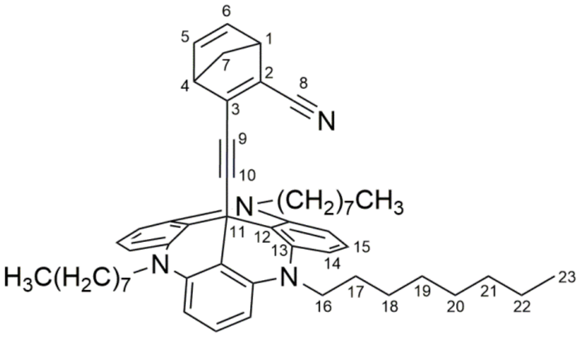

In THF (abs., 60 mL) 3-[2-(trimethylsilyl)ethynyl]bicyclo[2.2.1]hepta-2,5-diene-2-carbonitrile (**5**, 100 mg, 469 µmol) was dissolved under nitrogen atmosphere, octyl-TATA-BF_4 **6** [2] (397 mg, 562 µmol) and powdered potassium hydroxide (263 mg, 3.69 mmol) were added and the mixture was refluxed for 5 h. The mixture was poured onto saturated sodium chloride solution (50 mL) and the aqueous phase extracted with diethyl ether (3 × 50 mL). The combined organic layers were dried over magnesium sulfate and the solvent was removed under reduced pressure. The crude product was purified via column chromatography (aluminium oxide basic, diethyl ether) and recrystallized from ethanol to obtain an orange solid (222 mg, 292 µmol, 62%).

^{1}H NMR (600.1 MHz, C_6D_6, 298 K, TMS): δ = 7.21 (t, ^3J = 8.2 Hz, 3H, *H*-15), 6.61 (m, 6H, *H*-14), 5.91-5.86 (m, 1H, *H*-5), 5.76-5.73 (m, 1H, *H*-6), 3.86-3.80 (ps. t, 6H, *H*-16), 2.89-2.86 (m, 1H, *H*-4), 2.82-2.79 (m, 1H, *H*-1), 1.86-1.77 (m, 6H, *H*-17), 1.34-1.20 (m, 32H, H-7_a, H-7_b, *H*-18, *H*-19, *H*-20, *H*-21, *H*-22), 0.94-0.90 (ps. t, 9H, *H*-23) ppm.

^{13}C NMR (150.9 MHz, C_6D_6, 298 K, TMS): δ = 153.8 (s, *C*-2), 141.3 (s, *C*-5), 141.1 (s *C*-13), 140.8 (s, *C*-6), 129.4 (s, *C*-3), 129.1 (s, *C*-15), 109.4 (s, *C*-12), 105.6 (s, *C*-14), 79.5 (s, *C*-10), 72.2 (s, *C*), 56.4 (s, *C*-1), 53.6 (s, *C*-4), 47.1 (s, *C*-16), 32.2 (s, *C*), 30.2 (s, *C*-11), 29.8 (s, *C*), 29.7 (s, *C*-7), 27.2 (s, *C*), 25.9 (s, *C*-17), 23.1 (s, *C*-18), 14.4 (s, *C*-22) ppm.

MS (MALDI-TOF): m/z = 759.1 $[M]^+$.

IR (ATR): $\tilde{\nu}$ = 2953 (m), 2922 (m), 2851 (m), 2207 (w), 1617 (s), 1579 (vs), 1481 (s), 1456 (vs), 1394 (vs), 1372 (m), 1267 (m), 1246 (m), 1207 (w), 1167 (s), 1147 (m), 908 (w), 766 (vs), 731 (vs), 657 (w), 637 (m), 609 (w) cm^{-1}.

m.p. = 101.7 °C.

Elemental analysis calcd. (%) for $C_{53}H_{66}N_4$: C 83.86; H 8.76; N 7.38; found: C 83.53; H 8.65; N 7.32.

II.3 3-[2-Methyl-4-(trimethylsilylethynyl)phenyl]bicyclo[2.2.1]hepta-2,5-diene-2-carbonitrile (10).

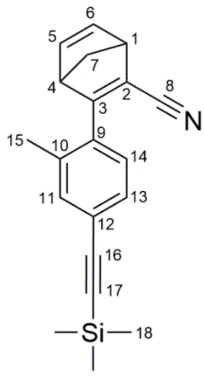

In a solution of toluene (13 mL), ethanol (3.75 mL) and H_2O (750 µL) 2-[2-methyl-4-[2-(trimethylsilyl)ethynyl]phenyl]-4,4,5,5-tetramethyl-1,3,2-dioxaborolane (**9** [3], 321 mg, 1.02 mmol), 3-bromo-bicyclo[2.2.1]hepta-2,5-diene-2-carbonitrile (**4** [1], 100 mg, 510 µmol), $Pd(PPh_3)_4$ (29.5 mg, 25.5 µmol) and sodium carbonate (136 mg, 1.28 mmol) were suspended under nitrogen atmosphere and refluxed for 12 h. To the mixture H_2O (10 mL) was added and the layers were separated. The water layer was extracted with dichloromethane (3 × 30 mL)

and the combined organic layers were dried over magnesium sulfate. The solvent was removed under reduced pressure and the crude product was purified via column chromatography (silica gel, cyclohexane/ethyl acetate, 4:1) to obtain a yellow oil (59.0 mg, 194 μmol, 38%).

^{1}H NMR (500.1 MHz, acetone-d$_6$, 298 K, TMS): δ = 7.39 (s, 1H, *H*-11), 7.33 (dd, ^3J = 8.0 Hz, ^4J = 1.0 Hz, 1H, *H*-13), 7.19 (d, ^3J = 8.0 Hz, 1H, *H*-14), 7.11-7.04 (m, 2H, *H*-5, *H*-6), 3.99-3.96 (m, 2H, *H*-1, *H*-4), 2.45 (td, ^3J = 6.9 Hz, ^4J = 1.6 Hz, 1H, *H*-7$_a$), 2.32 (s, 3H, *H*-9), 2.21 (td, ^3J = 6.9 Hz, ^4J = 1.6 Hz, 1H, *H*-7$_b$), 0.24 (s, 9H, *H*-18) ppm.

^{13}C NMR (125.8 MHz, acetone-d$_6$, 298 K, TMS): δ = 174.1 (s, *C*-3), 144.0 (s, *C*), 142.6 (s, *C*), 137.0 (s, C-10), 135.9 (s, *C*-9), 134.8 (s, *C*-11), 130.0 (s, *C*-13), 128.2 (s, *C*-14), 124.7 (s, *C*-12), 123.6 (s, *C*-2), 105.5 (s, *C*-16), 95.7 (s, *C*-17), 73.7 (s, *C*-7), 58.3 (s, *C*), 55.4 (s, *C*), 20.4 (s, *C*-9), 0.0 (s, *C*-18) ppm.

^{29}Si NMR (99.4 MHz, acetone-d$_6$, 298 K, TMS): δ = -17.48 ppm.

MS (EI, 70eV): m/z = 303.14 [M]$^+$.

IR: $\tilde{\nu}$ = 2958 (br, w), 2204 (m), 2151 (w), 1606 (w), 1560 (w), 1493 (w), 1450 (w), 1310 (w), 1295 (m), 1233 (w), 1004 (w), 949 (w), 899 (w), 834 (vs), 814 (s), 759 (m), 723 (vs), 658 (m) cm^{-1}.

HRMS (EI, 70 eV): m/z [M]$^+$ calcd. for $C_{20}H_{21}NSi$: 303.14433, found: 303.14410.

II.4 12c-(4-(2-Cyanobicyclo[2.2.1]hepta-2,5-diene-3-yl)-3-methylphenyl)ethynyl-4,8,12-tri-*n*-octyl-4,8,12-triazatriangulene (2).

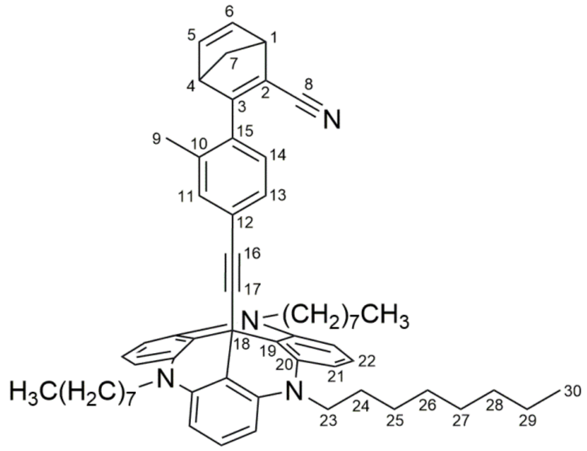

In THF (abs., 40 mL) 3-[2-methyl-4-(trimethylsilylethynyl)phenyl]bicyclo[2.2.1]hepta-2,5-diene-2-carbonitrile (**10**, 65.0 mg, 214 μmol) was dissolved under nitrogen atmosphere and octyl-TATA-BF$_4$ **6** [2] (181 mg, 257 μmol) and powdered potassium hydroxide (95.9 mg, 1.71 mmol) were added and the mixture and was refluxed for 1 h. The mixture was poured onto sat. sodium chloride solution (30 mL) and extracted with diethyl ether (3 × 50 mL). The combined organic layers were dried over magnesium sulfate and the solvent was removed under reduced pressure. The crude product was purified via column chromatography (aluminium oxide basic, diethyl ether) and recrystallized from ethanol to obtain a grey solid (80.0 mg, 94.2 μmol, 44%).

¹H NMR (500.1 MHz, C_6D_6, 298 K, TMS): δ = 7.25 (t, ^3J = 8.3 Hz, 3H, *H*-22), 6.85 (dd, ^3J = 8.1 Hz, ^4J = 1.1 Hz, 1H, *H*-13), 6.82 (s, 1H, *H*-11), 6.66-6.61 (m, 7H, *H*-21, H-14), 6.33 (dd, ^3J = 5.1 Hz, ^3J = 3.0 Hz, 1H, *H*-5), 6.19 (dd, ^3J = 5.1 Hz, ^3J = 3.0 Hz, 1H, *H*-6), 3.84-3.78 (ps. t, 6H, *H*-23), 3.24-3.21 (m, 1H, *H*-4), 3.02-2.99 (m, 1H, *H*-1), 1.85-1.77 (m, 6H, *H*-24), 1.76 (s, 3H, *H*-9), 1.64 (td, ^3J = 6.8 Hz, ^4J = 1.5 Hz, 1H, H-7_a), 1.54 (td, ^3J = 6.8 Hz, ^4J = 1.5 Hz, 1H, H-7_b), 1.31-1.15 (m, 30H, *H*-25, *H*-26, *H*-27, *H*-28, *H*-29), 0.91 (ps. t, 9H, *H*-30) ppm.

¹³C NMR (125.8 MHz, C_6D_6, 298 K, TMS): δ = 172.3 (s, *C*-3), 142.9 (s, *C*-5), 141.2 (s, *C*-20), 141.0 (s, *C*-6), 135.0 (s, *C*-10), 134.5 (s, *C*-11), 133.5 (s, *C*-15), 129.4 (s, *C*-13), 128.7 (s, *C*-22) 126.7 (s, *C*-14), 125.0 (s, *C*-12), 122.2 (s, *C*-2), 111.1 (s, *C*-19), 105.7 (s, *C*-21), 95.6 (s, *C*-16), 84.1 (s, *C*-17), 72.4 (s, *C*-7), 57.2 (s, *C*-1), 54.5 (s, *C*-4), 46.7 (s, *C*-23), 32.2 (s, *C*-27), 29.7 (s, *C*-28), 29.7 (s, *C*-29), 29.1 (s, *C*-18), 27.2 (s, *C*-25), 26.2 (s, *C*-24), 23.0 (s, *C*-26), 20.0 (s, *C*-9), 14.4 (s, *C*-30) ppm.

MS (MALDI-TOF): m/z = 849.4 $[M]^+$.

IR: $\tilde{\nu}$ = 2922 (s), 2852 (m), 2204 (w), 1615 (s), 1579 (vs), 1482 (vs), 1456 (vs), 1393 (cs), 1373 (m), 1293 (w), 1267 (m), 1244 (m), 1167 (s), 1022 (w), 911 (w), 886 (w), 828 (w),, 816 (w), 789 (w), 772 (m), 748 (m), 724 (s), 696 (vs), 657 (w), 608 (w) cm^{-1}.

m.p. = 73.6 °C.

Elemental analysis calcd. (%) for $C_{60}H_{72}N_4$: C 84.86; H 8.55; N 6.60; found: C 84.63; H 8.48; N 6.57.

II.5 Synthesis of 4,8,12-Trioxatriangulenium tetrakis[3,5-bis(trifluoromethyl)phenyl]-borate (8)

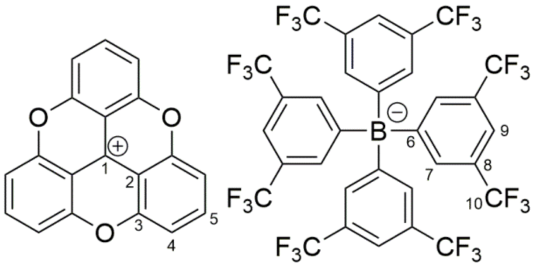

In dichloromethane (200 mL) 4,8,12-trioxatriangulenium tetrafluoroborate (**7** [4], 636 mg, 1.71 mmol) and sodium tetrakis[3,5-bis(trifluoromethyl)phenyl]borate (1.89 g, 2.10 mmol) were suspended and stirred at room temperature for 2 h. The mixture was filtered and the solution was washed with water (3 × 150 mL) and dried over magnesium sulfate. The solvent was removed under reduced pressure and the crude product was dissolved in 25 mL ethyl acetate and precipitated by adding 400 mL cyclohexane. Filtration gave 1.77 g (1.54 mmol, 91%) of a yellowish solid.

¹H NMR (500.1 MHz, acetone-d_6, 298 K, TMS): δ = 8.66 (t, ^3J = 8.5 Hz, 3H, *H*-5), 7.99 (d, ^3J = 8.5 Hz, 6H, *H*-4), 7.79 (t, ^4J = 2.5 Hz, 8H, *H*-7), 7.67 (s, 4H, *H*-9) ppm.

¹³C NMR (125.8 MHz, acetone-d_6, 298 K, TMS): δ = 162.6 (q, *C*-6), 154.7 (s, *C*-3), 144.7 (m, *C*-5), 135.5 (m, *C*-7), 130.0 (m, *C*-10), 125.4 (d, *C*-8), 118.4 (m, *C*-9), 113.6 (s, *C*-4), 107.3 (s, *C*-2) ppm.

^{19}F NMR (470 MHz, acetone-d_6, 298 K, TMS): δ = -62.2 ppm.
^{11}B NMR (160 MHz, acetone-d_6, 298 K, TMS): δ = -5.86 ppm.

IR (ATR): $\tilde{\nu}$ = 2311 (w), 2164 (w), 1635 (s), 1552 (m), 1467 (m), 1355 (s), 1275 (s), 1143 (s), 1112 (s), 1063 (s), 1021 (s), 900 (m), 887 (m), 776 (s), 681 (s), 558 (s), 412 (m) cm^{-1}.

MS (ESI, pos): m/z = 285.05 $[C_{19}H_9O_3]^+$.

MS (ESI, neg): m/z = 863.07 $[C_{32}H_{12}BF_{24}]^-$.

m.p. = 202 °C.

HRMS (ESI, pos): m/z $[M]^+$ calc. for $C_{19}H_9O_3$: 285.05462, found: 285.05428.
(ESI, neg): m/z $[M]^-$ calc. for $C_{32}H_{12}BF_{24}$: 862.06906, found: 862.06892.

II.6 12c-(2-Cyanobicyclo[2.2.1]hepta-2,5-diene-3-yl)-4,8,12-trioxatriangulene (3).

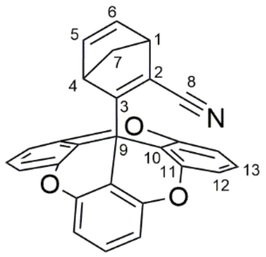

In THF (abs., 15 mL), 3-bromobicyclo[2.2.1]hepta-2,5-diene-2-carbonitrile (**4** [5-7], 213 mg, 1.09 mmol) was dissolved under nitrogen atmosphere und the solution was cooled to −78 °C. To the solution *n*-BuLi (436 µL, 1.09 mmol, 2.5 M in *n*-hexane) was added slowly and stirred for 45 min. 4,8,12-Trioxatriangulenium tetrakis[3,5-bis(trifluormethyl)phenyl]borate (**8**, 1.38 g, 1.20 mmol), dissolved in THF (abs., 30 mL), was added slowly and stirred for 45 min at −78 °C and further for 20 h at room temperature. To the solution, diethyl ether (30 mL) was added and the solution was washed with water (3 × 50 mL). The combined organic layers were dried over magnesium sulfate and the solvent was removed under reduced pressure. The crude product was purified via column chromatography (alox basic, diethyl ether) and recrystallized from methanol to obtain a colorless solid (147 mg, 367 µmol, 22%).

^{1}H NMR (500.1 MHz, C_6D_6, 298 K, TMS): δ = 6.93 (t, ^3J = 8.3 Hz, 3H, *H*-13), 6.87-6.81 (m, 6H, *H*-12), 6.01 (dd, ^3J = 5.0 Hz, ^3J = 3.1 Hz, 1H, *H*-5), 5.83 (dd, ^3J = 5.0 Hz, ^3J = 3.1 Hz, 1H, *H*-6), 3.55-3.53 (m, 1H, *H*-4), 3.03-3.00 (m, 1H, *H*-1), 1.25-1.20 (m, 2H, *H*-7) ppm.

^{13}C NMR (125.8 MHz, C_6D_6, 298 K, TMS): δ = 173.7 (s, *C*-3), 153.3 (d, *C*-11), 142.3 (s, *C*-5), 140.3 (s, *C*-6), 129.9 (s, *C*-13), 120.4 (s, *C*-2), 111.9 (d, *C*-12), 109.9 (s, *C*-10), 70.7 (s, *C*-7), 55.4 (s, *C*-1), 52.6 (s, *C*-4), 31.3 (s, *C*-9) ppm.

MS (EI, 70eV): m/z = 401.07 $[M]^+$.

IR (ATR): $\tilde{\nu}$ = 2946 (w), 2202 (w), 1743 (w), 1612 (s), 1481 (m), 1456 (s), 1306 (w), 1260 (vs), 1065 (m), 1041 (m), 1010 (vs), 934 (w), 903 (m), 877 (m), 786 (m), 773 (m), 688 (m), 597 (w), 576 (w) cm^{-1}.

HRMS (EI, 70 eV): m/z $[M]^+$ calcd. for $C_{27}H_{15}NO_3$: 401.10519, found: 401.10515.

III. NMR spectra

III.1 3-[2-(Trimethylsilyl)ethynyl]bicyclo[2.2.1]hepta-2,5-diene-2-carbonitrile (5).

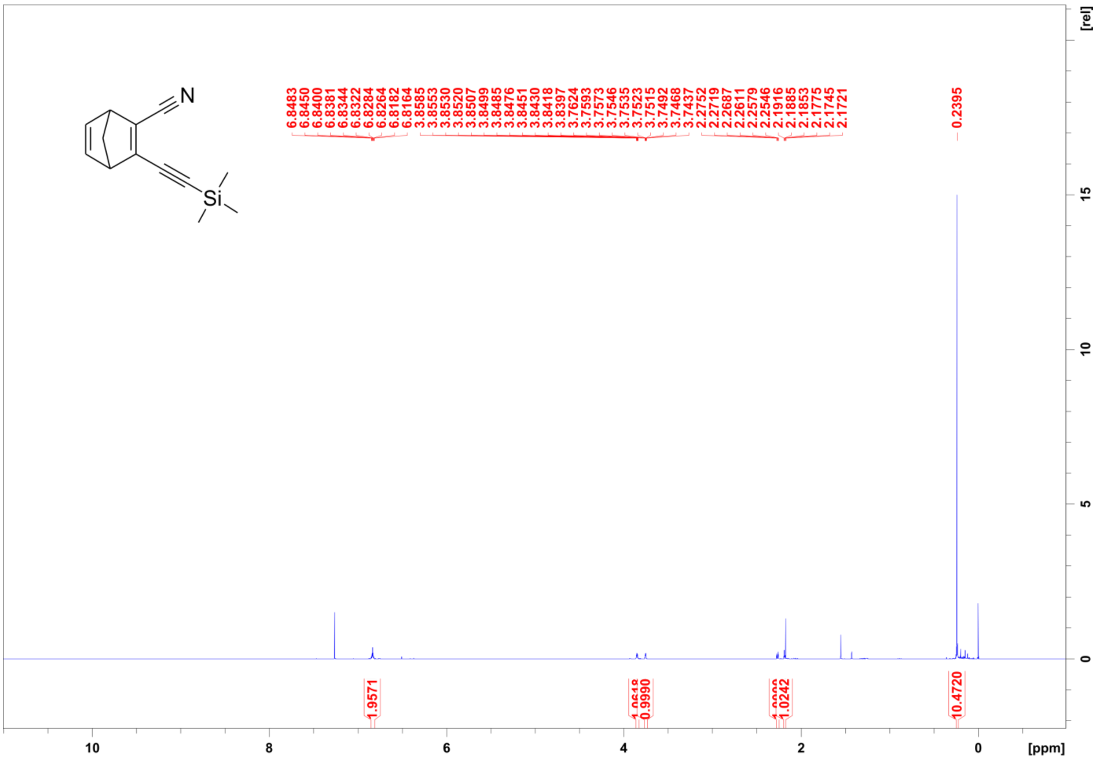

Figure S1. ^{1}H NMR spectrum (500.1 MHz, $CDCl_3$) of compound **5**.

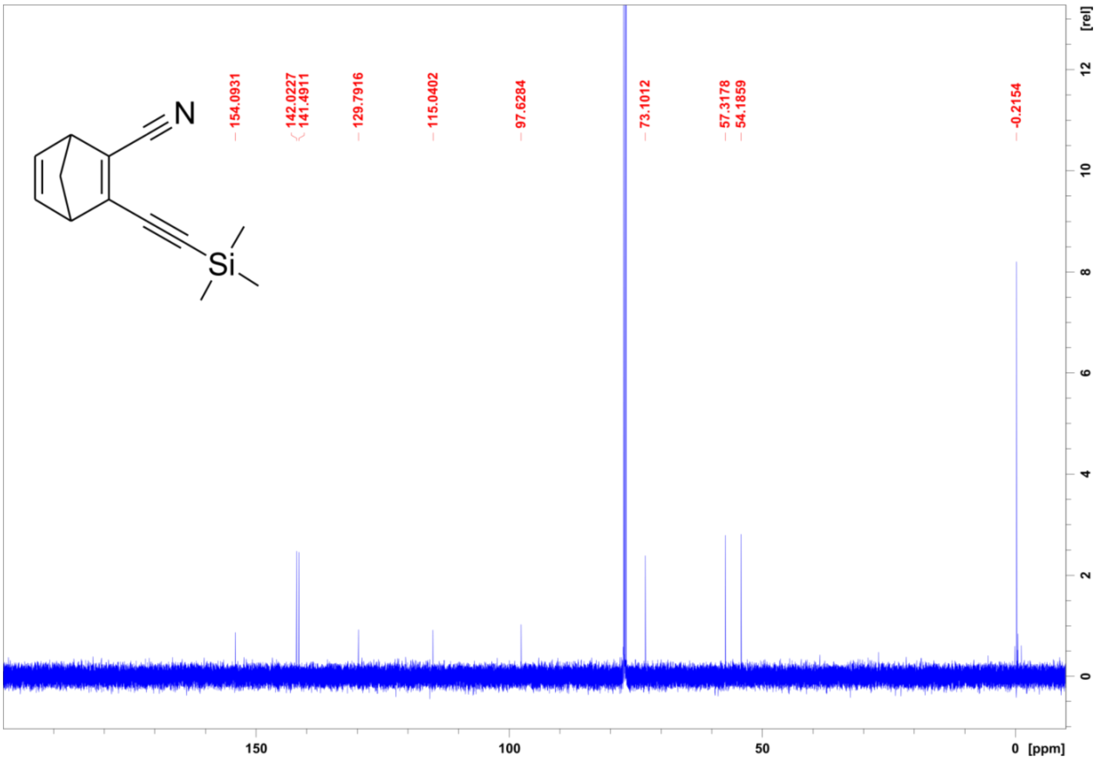

Figure S2. ^{13}C NMR spectrum (125.8 MHz, $CDCl_3$) of compound **5**.

III.2 12c-(2-(2-Cyanobicyclo[2.2.1]hepta-2,5-diene-3-yl)ethynyl)-4,8,12-tri-*n*-octyl-4,8,12-triazatriangulene (1).

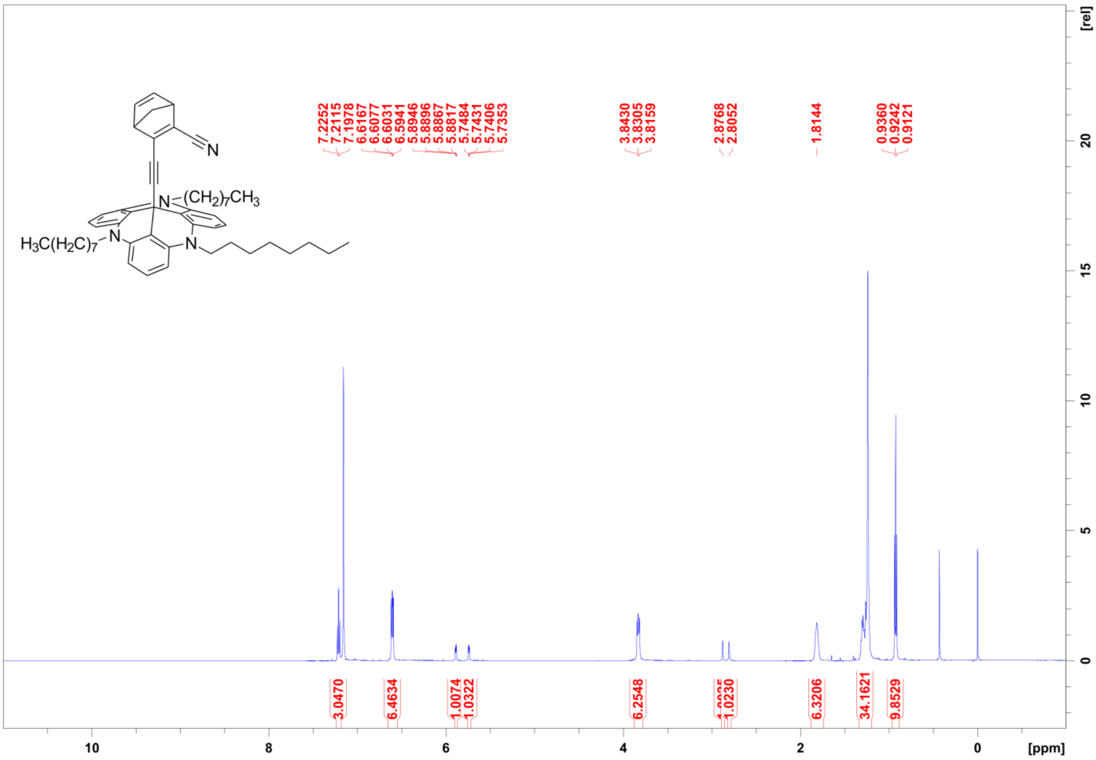

Figure S3. ^{1}H NMR spectrum (600.1 MHz, C_6D_6) of compound **1**.

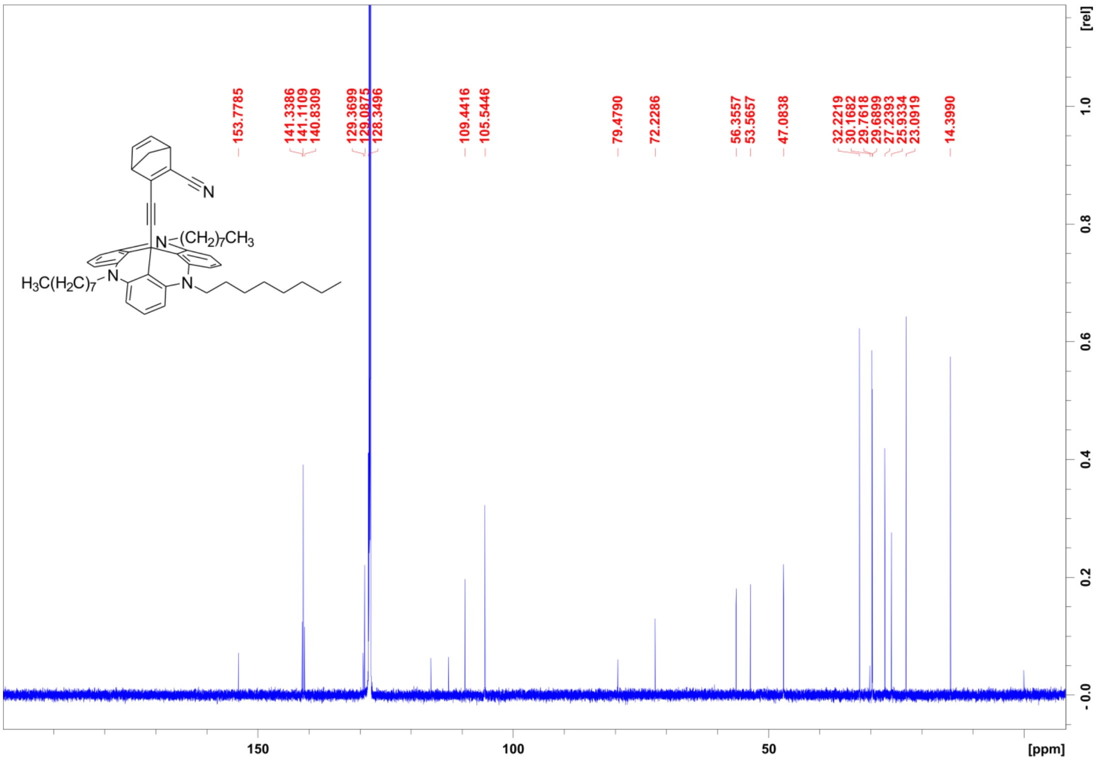

Figure S4. ^{13}C NMR spectrum (150.9 MHz, C_6D_6) of compound **1**.

III.3 3-[2-Methyl-4-(trimethylsilylethynyl)phenyl]bicyclo[2.2.1]hepta-2,5-diene-2-carbonitrile (10).

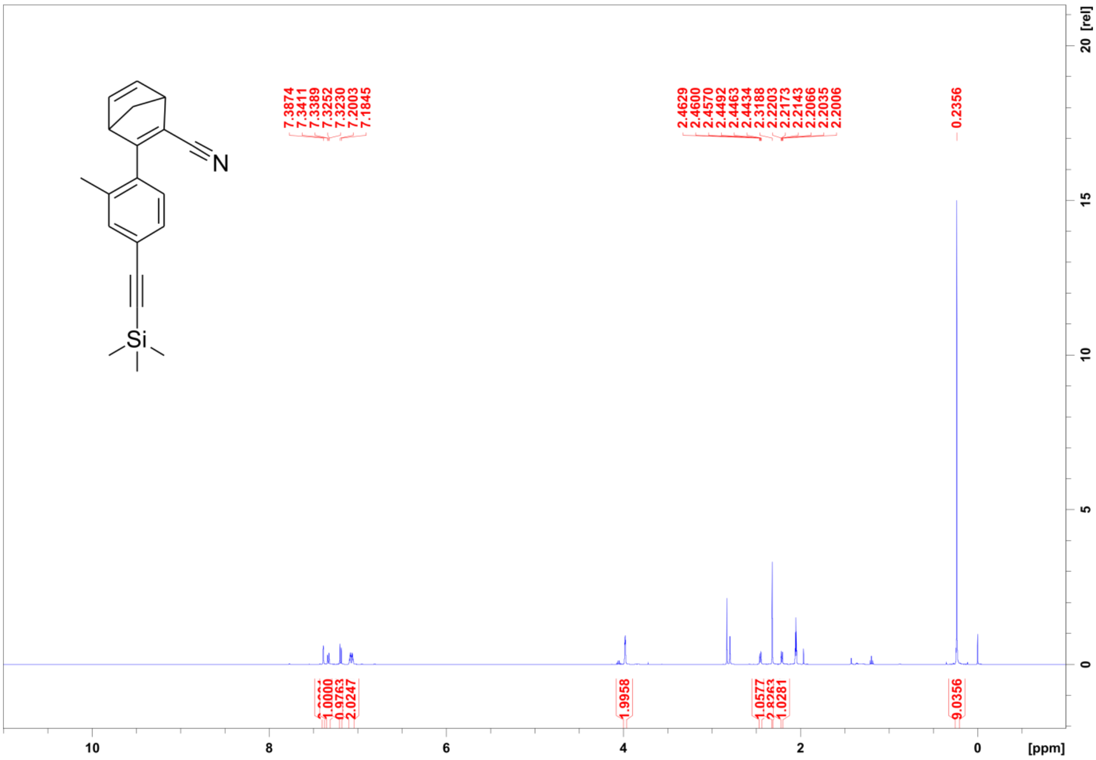

Figure S5. ^{1}H NMR spectrum (500.1 MHz, acetone-d_6) of compound **10**.

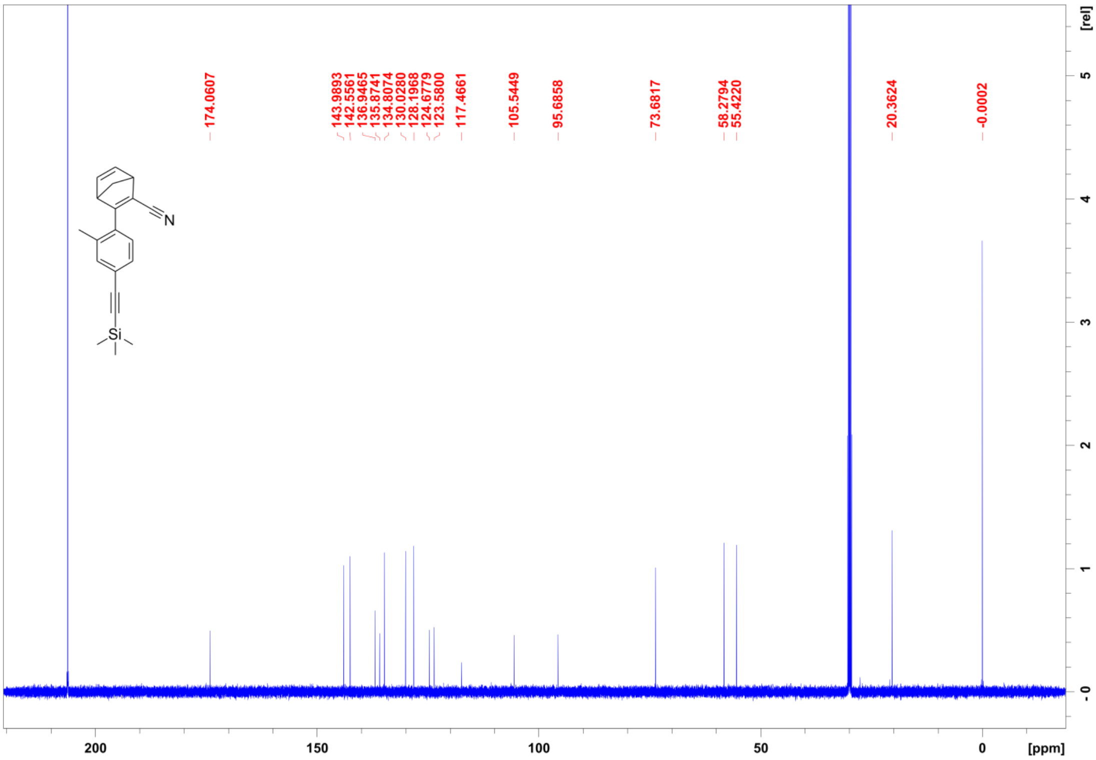

Figure S6. ^{13}C NMR spectrum (125.8 MHz, acetone-d_6) of compound **10**.

III.4 12c-(4-(2-Cyanobicyclo[2.2.1]hepta-2,5-diene-3-yl)-3-methylphenyl)ethynyl-4,8,12-tri-*n*-octyl-4,8,12-triazatriangulene (2).

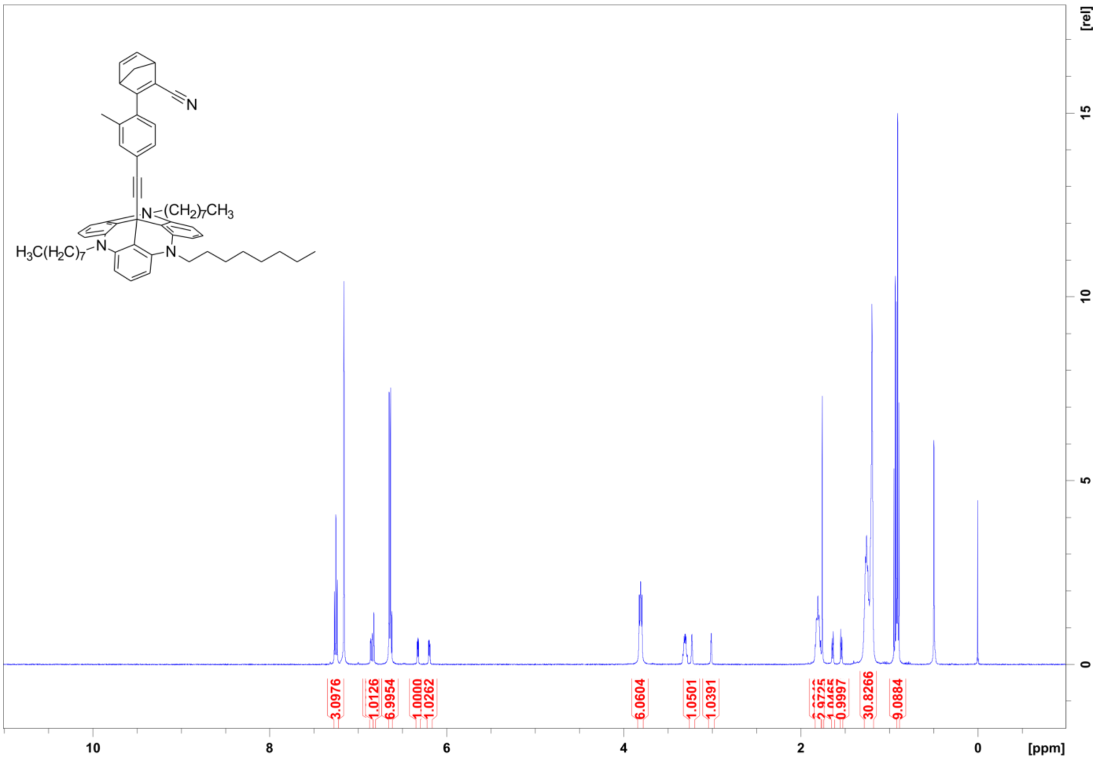

Figure S7. ^{1}H NMR spectrum (500.1 MHz, C_6D_6) of compound **2**.

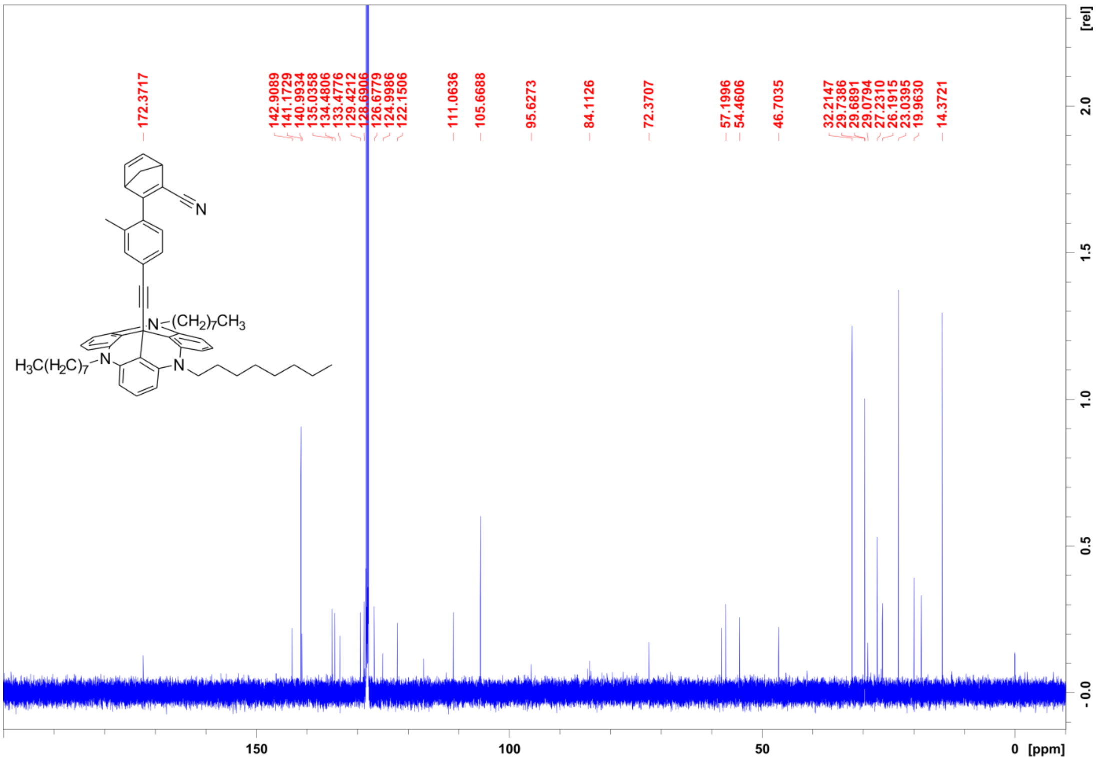

Figure S8. ^{13}C NMR spectrum (125.8 MHz, C_6D_6) of compound **2**.

III.5 Synthesis of 4,8,12-trioxatriangulenium tetrakis[3,5-bis(trifluoromethyl)phenyl]-borate (8)

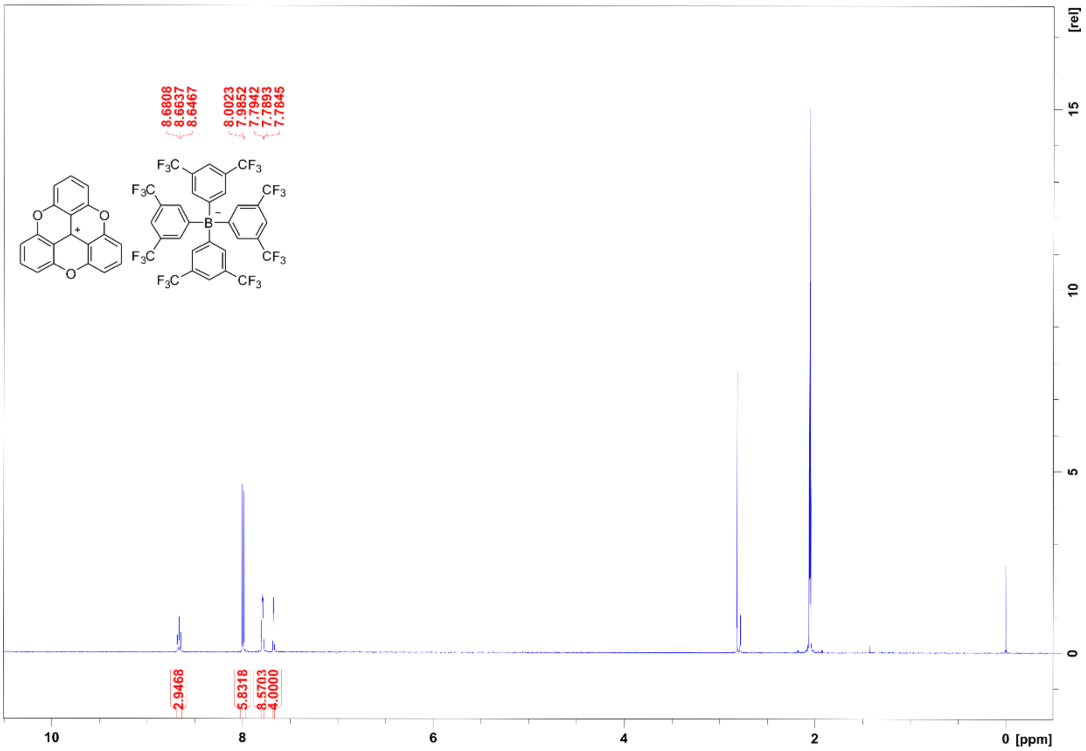

Figure S9. ^{1}H NMR spectrum (500.1 MHz, acetone-d_6) of compound **8**.

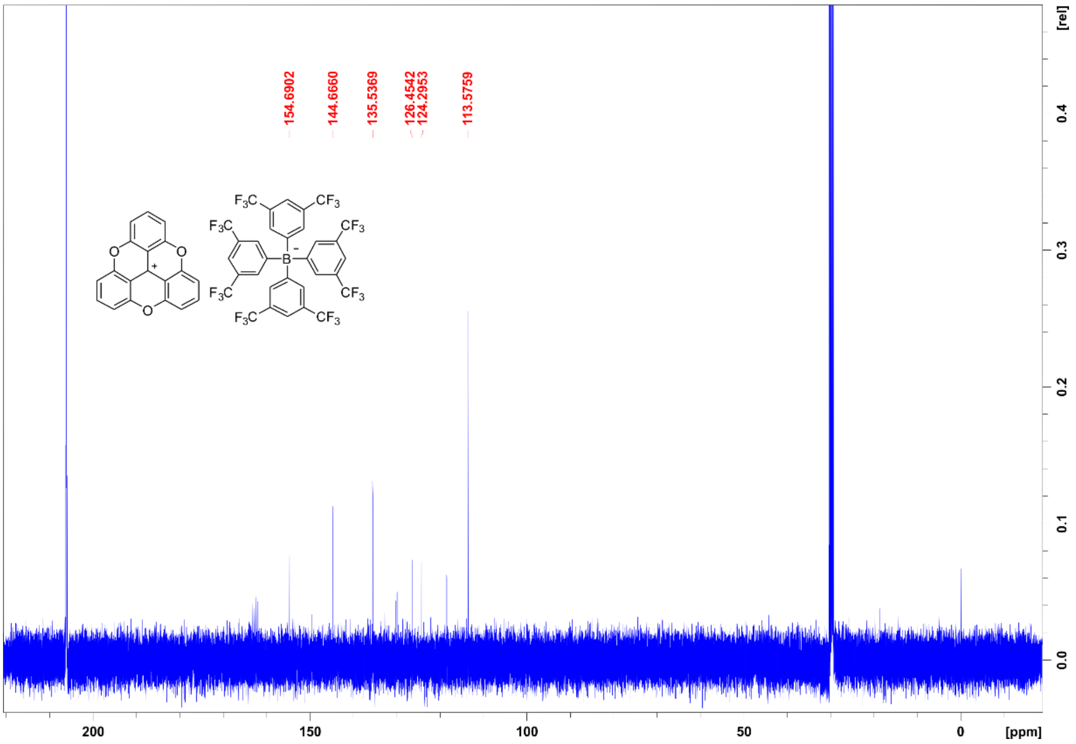

Figure S10. ^{13}C NMR spectrum (125.8 MHz, acetone-d_6) of compound **8**.

III.6 12c-(2-Cyanobicyclo[2.2.1]hepta-2,5-diene-3-yl)-4,8,12-trioxatriangulene (3).

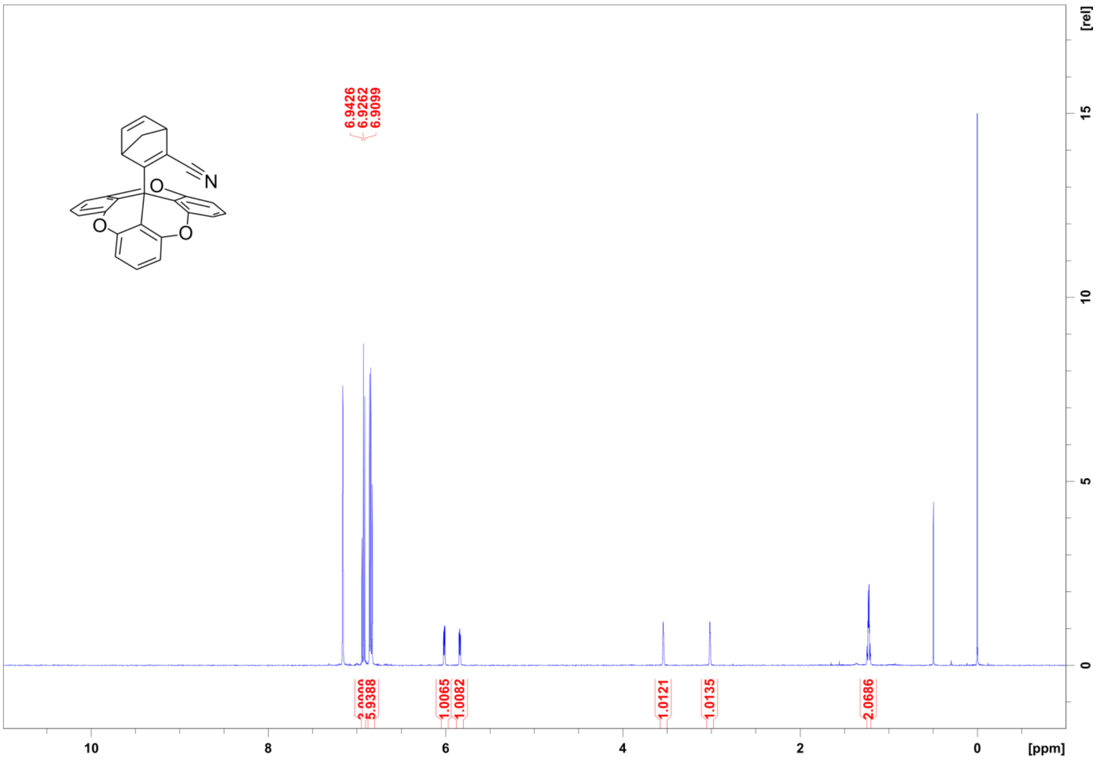

Figure S11. ^{1}H NMR spectrum (500.1 MHz, acetone-d_6) of compound **3**.

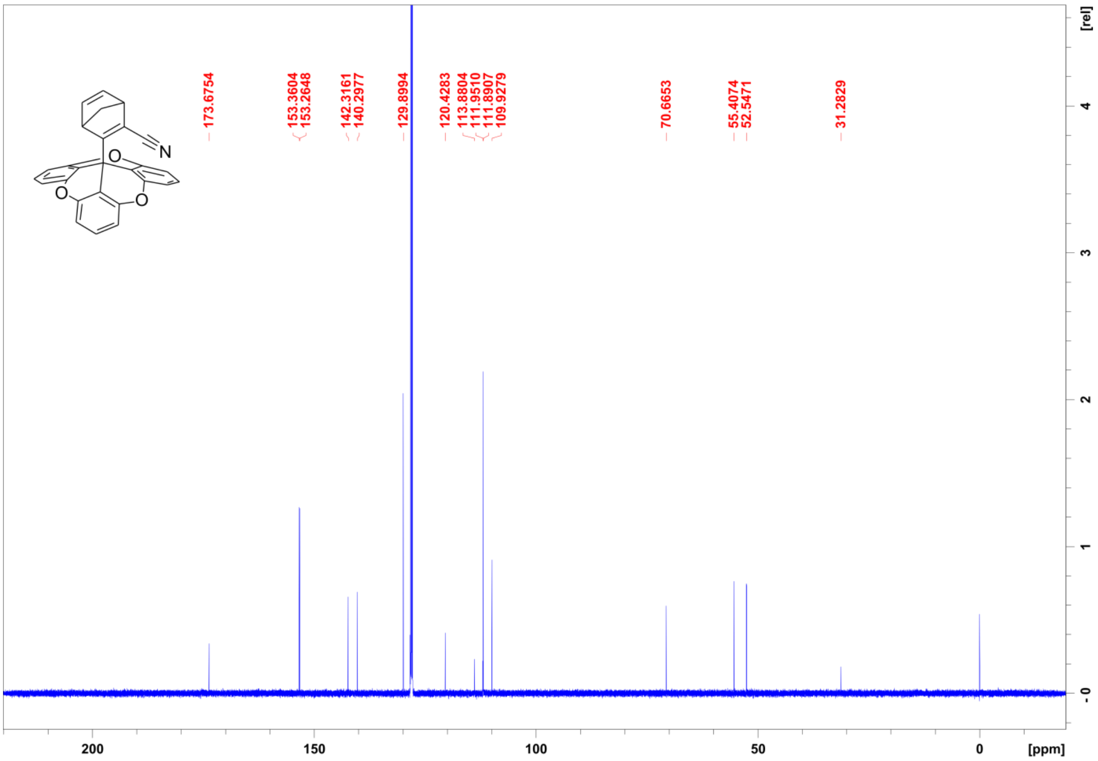

Figure S12. ^{13}C NMR spectrum (125.8 MHz, acetone-d_6) of compound **3**.

IV. UV–vis absorption spectra

IV.1 Methods

UV–vis spectra were recorded on a PerkinElmer Lambda 650 Photospectrometer in a 1 cm path length quartz cuvette. Irradiation of UV–vis samples were carried out at 25 °C using a self-built LED positioned at a distance of 1 cm from the sample.

IV.2 UV/Vis spectra

Compound **1**:

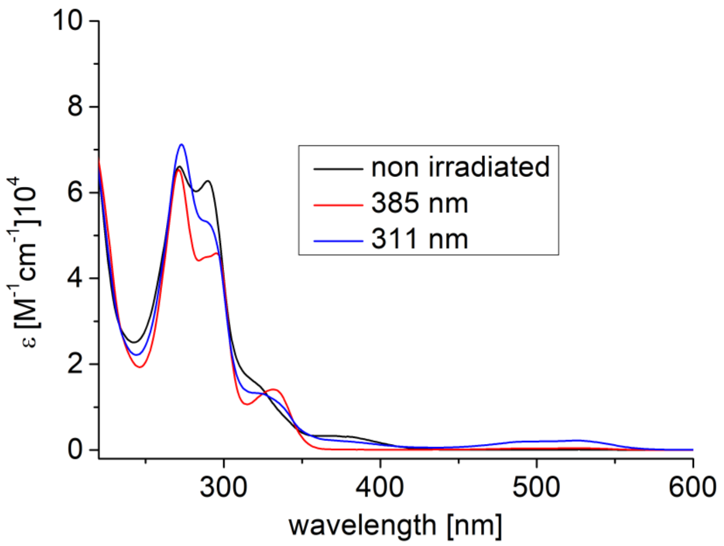

Figure S13. UV–vis spectra of compound **1** in THF at rt (31.6 μmol/L). Upon irradiation with 385 nm the [2 + 2] cycloaddition and with 311 nm the [2 + 2] cycloreversion take place with partly decomposition of **1**.

Compound **2**:

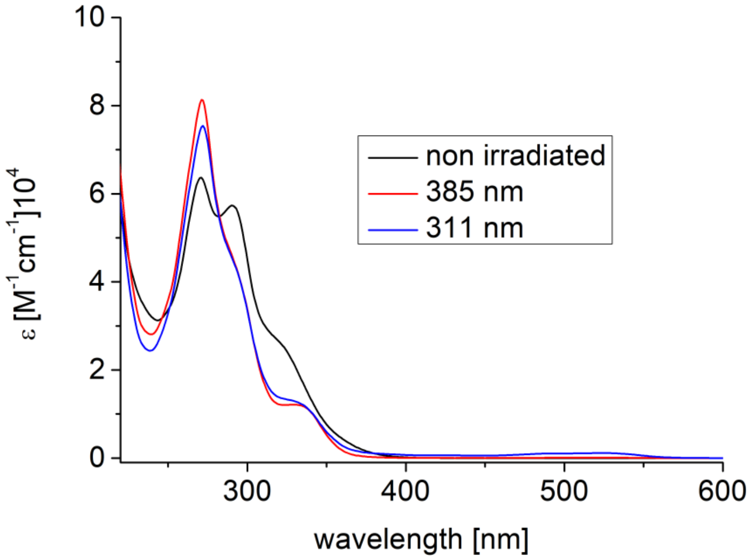

Figure S14. UV–vis spectra of compound **2** in THF at rt (30.6 μmol/L). Upon irradiation with 385 nm the [2 + 2] cycloaddition and with 311 nm the [2 + 2] cycloreversion take place with partly decomposition of **2**.

Compound **3**:

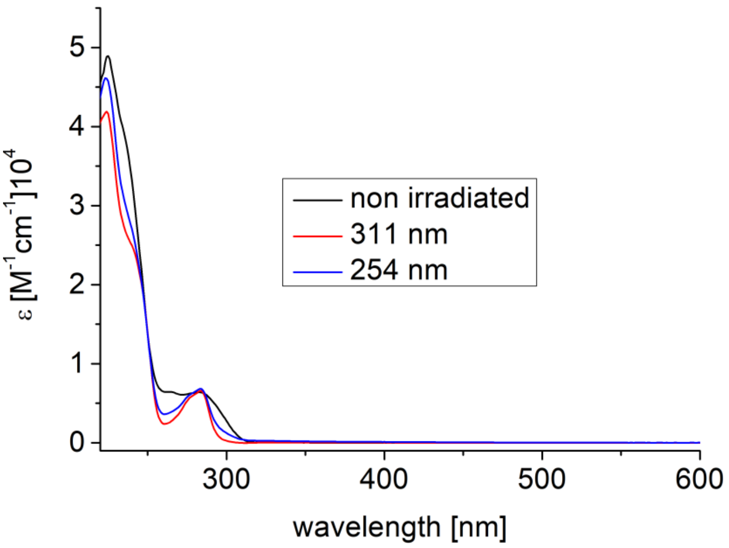

Figure S15. UV–vis spectra of compound **3** in THF at rt (79.7 μmol/L). Upon irradiation with 311 nm the [2 + 2] cycloaddition and with 254 nm the [2 + 2] cycloreversion take place.

V. Kinetic studies in solution by ^{1}H NMR spectroscopy

V.1 Thermal isomerization rate measurements by ^{1}H NMR

V.1.1 Compound 1: 12c-(2-(2-cyanobicyclo[2.2.1]hepta-2,5-diene-3-yl)ethynyl)-4,8,12-tri-*n*-octyl-4,8,12-triazatriangulene

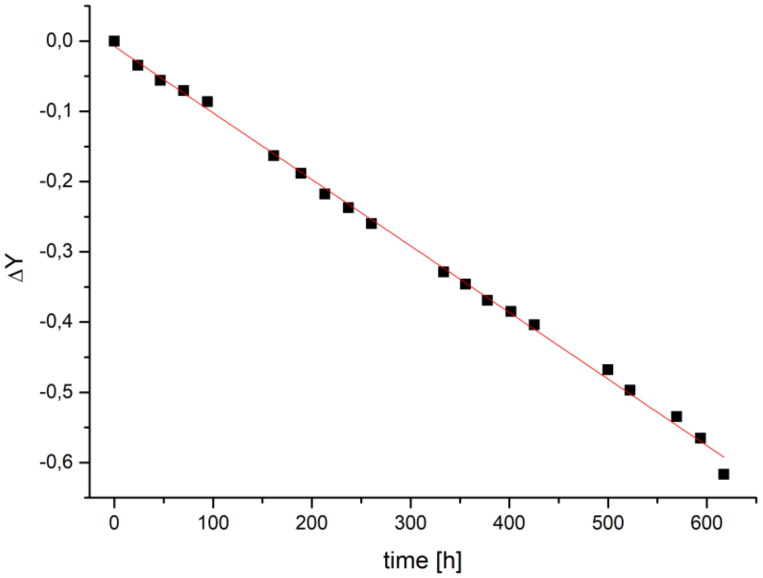

Figure S16. Determination of the thermal isomerization rate *k* of **1b** (QC) by ^{1}H NMR spectroscopy (toluene, 294.5 K, 800 µmol/L, under nitrogen). ΔY: ln { $[QC]_t/[QC]_0$ }, $[QC]_t$: ^{1}H NMR integral of the CH_2 group neighbouring the N bridge atom of the TATA platform in QC **1b** at time *t*, $[QC]_0$ corresponding ^{1}H integral at t = 0. A rate constant of $k = 0.95 \cdot 10^{-3}$ s^{-1} was determined from a linear fit of the $\Delta Y/t$ curve. The half-life of **1b** at 293.5 K in toluene was determined as 742.7 h.

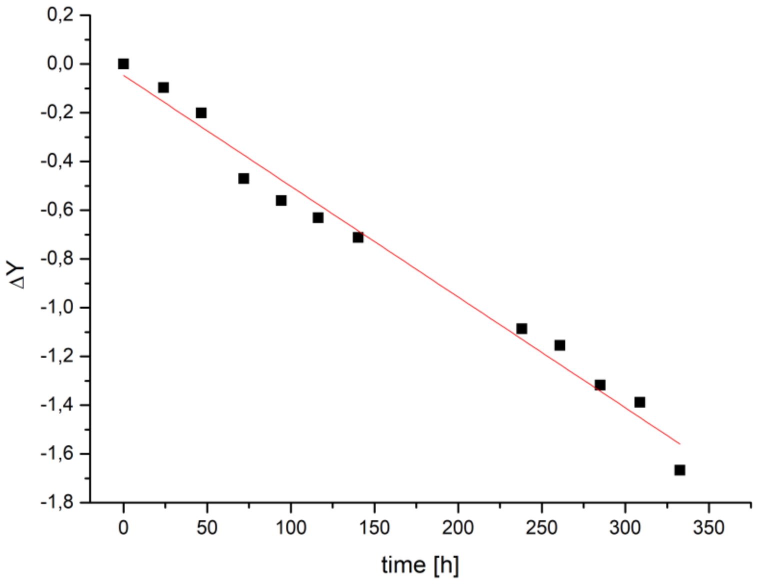

Figure S17. Determination of the thermal isomerization rate *k* of **1b** (QC) by ^{1}H NMR spectroscopy (toluene, 306 K, 800 µmol/L, under nitrogen). ΔY: ln { $[QC]_t/[QC]_0$ }, $[QC]_t$: ^{1}H NMR integral of the CH_2 group neighbouring the N bridge atom of the TATA platform in QC **1b** at time *t*, $[QC]_0$ corresponding ^{1}H integral at $t = 0$. A rate constant of $k = 4.55 \cdot 10^{-3}$ s^{-1} was determined from a linear fit of the ΔY/*t* curve. The half-life of **1b** at 305 K in toluene was determined as 152.3 h.

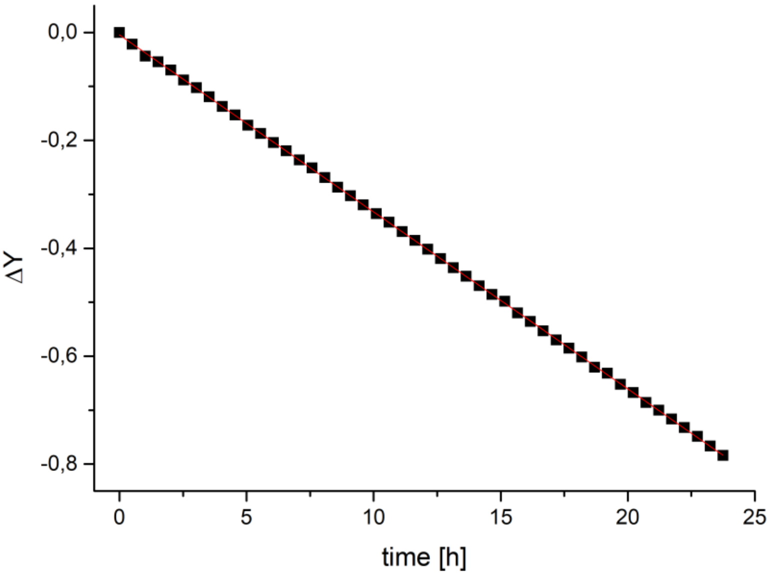

Figure S18. Determination of the thermal isomerization rate *k* of **1b** (QC) by ^{1}H NMR spectroscopy (toluene, 318 K, 800 µmol/L, under nitrogen). ΔY: ln { $[QC]_t/[QC]_0$ }, $[QC]_t$: ^{1}H NMR integral of the CH_2 group neighbouring the N bridge atom of the TATA platform in QC **1b** at time *t*, $[QC]_0$ corresponding ^{1}H integral at $t = 0$. A rate constant of $k = 3.28 \cdot 10^{-2}$ s^{-1} was determined from a linear fit of the ΔY/*t* curve. The half-life of **1b** at 316 K in toluene was determined as 21.1 h.

V.2 Arrhenius Plots for compound 1 in solution

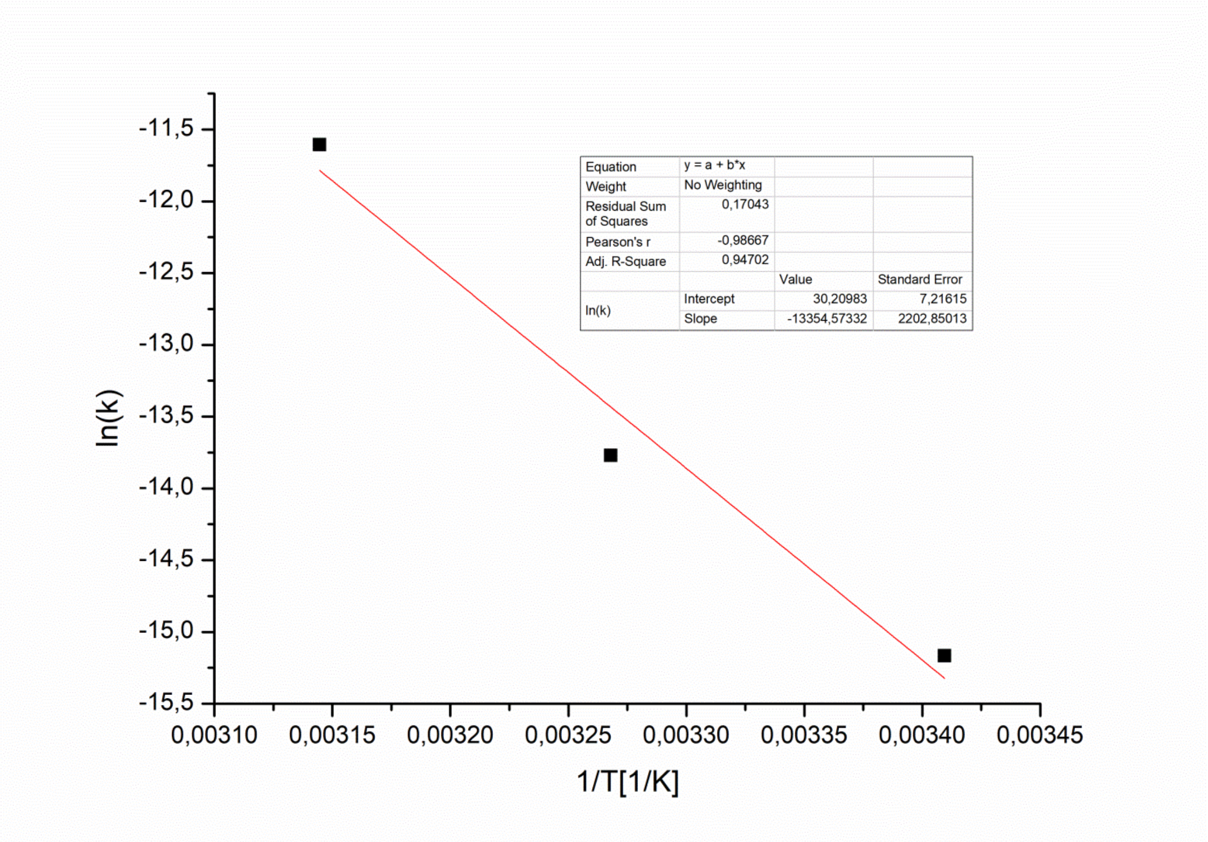

Figure S19. Arrhenius plot of the QC→NB isomerization of compound **1** which shows an activation energy of 111 kJ/mol.

VI. STM measurements

SAMs were prepared by immersing an Au(111) single crystal 3 hours in a 1–100 µM solution of **1** in toluene at room temperature. Afterwards the samples were rinsed with toluene and dried in the air. STM measurements were performed under ambient conditions, using a PicoPlus SPM (Agilent) and Pt/Ir tips.

VII. Calculations

General

All geometry optimizations were carried out using density functional theory with the Minnesota functional M06-2X [8] in cooperation with Grimmes D3 [9] dispersion correction and the large triple zeta basis def2-TZVP [10]. This level performed well in Grimme's study on basic properties of a selected data base of structures [11]. The calculations were carried out with Turbomole7.2 [12], the m4 grid (in Turbomole nomenclature) and resolution-of-identity (RI) with multipole accelerated RI-J (marij). All stationary points were characterized by frequency calculations.

Coordinates

1a Norbornadiene-ethinyl-TATA

$E_{M062x\text{-}D3/def2TZVPP}$ = -1334.818694137

Nimag = 2 (-17.61 cm^{-1}; -6.98 cm^{-1})

C	0.2951221	-0.0928872	-0.1011107
C	-1.1704890	0.0583325	0.0307701
C	-1.8459803	-1.2871414	-0.0863013
C	-1.4660755	0.6715255	1.3802029
C	-1.6505093	0.9771099	-1.0692086
C	-2.0166023	0.4470018	-2.3024972
C	-2.3089692	1.2999839	-3.3661180
C	-2.2386146	2.6708262	-3.1696166
C	-1.9128988	3.2121107	-1.9358891
C	-1.6242241	2.3558268	-0.8734252
C	-1.6581455	-0.1500244	2.4865432
C	-1.4436158	2.0566046	1.5252156
C	-1.5573038	2.6223135	2.7950065
C	-1.7095793	1.7905484	3.8931360
C	-1.7761609	0.4122027	3.7570676
H	-1.5258145	3.6970613	2.9141541
H	-1.7950422	2.2281439	4.8787235
H	-1.9156187	-0.2236005	4.6214454
H	-1.8804372	4.2837995	-1.7919981
H	-2.5840991	0.8914577	-4.3295204
H	-2.4584959	3.3342520	-3.9952168
C	-2.2135302	-1.7702573	-1.3397407
C	-2.7092283	-3.0680069	-1.4664217
C	-2.8470960	-3.8518472	-0.3315202
C	-2.5305116	-3.3665867	0.9278284
C	-2.0351589	-2.0682900	1.0509749
H	-2.9850852	-3.4502756	-2.4404461
H	-3.2290342	-4.8592200	-0.4288256
H	-2.6682762	-3.9798697	1.8085969
N	-1.7190682	-1.5269515	2.2918696
N	-2.0723133	-0.9366409	-2.4432285
N	-1.3062321	2.8403748	0.3878158
H	-1.3325165	3.8377176	0.5145511
H	-2.4407027	-1.2816335	-3.3135604
H	-1.9634605	-2.0799268	3.0960594
C	5.1902861	0.1449561	-0.3944998
C	3.7945606	0.7798825	-0.2757764
C	2.8939936	-0.2162297	-0.3304604
C	3.6995631	-1.5188465	-0.4949560
C	4.4984532	-1.7012154	0.8018948
C	5.3851804	-0.7160310	0.8609508
C	4.8322591	-0.9762285	-1.4018125
H	3.1192811	-2.3681946	-0.8372177
H	4.4734997	-0.5956125	-2.3564633
H	5.6385924	-1.6930174	-1.5405454
C	3.5404447	2.1563930	-0.0592153
N	3.3664019	3.2799138	0.1124753
C	1.4937530	-0.1463250	-0.2039334
H	4.2997123	-2.4654899	1.5367172
H	6.0798673	-0.4901947	1.6549143
H	5.9899191	0.8322061	-0.6473880

1b Quadricyclane-ethinyl-TATA

$E_{M062x\text{-}D3/def2TZVPP}$ = -1334.790048271

Nimag = 2 (-17.69 cm^{-1}; -16.54 cm^{-1})

C	0.3054503	0.3034247	-0.0945918
C	-1.1302770	-0.0443125	-0.0006913
C	-1.9801978	1.1980518	-0.1257588
C	-1.4616538	-1.0110023	-1.1140504
C	-1.3719566	-0.7026080	1.3381671
C	-1.6973779	0.0753641	2.4446918
C	-1.7628444	-0.5075456	3.7096545
C	-1.5059887	-1.8641665	3.8408492
C	-1.2152576	-2.6576889	2.7424688
C	-1.1582689	-2.0717652	1.4776801
C	-1.8709972	-0.5278849	-2.3527081
C	-1.2454623	-2.3740050	-0.9257332
C	-1.3857235	-3.2533699	-1.9996441
C	-1.7589621	-2.7531021	-3.2371711
C	-2.0180939	-1.4040318	-3.4274294
H	-1.2041283	-4.3108295	-1.8608406
H	-1.8658301	-3.4336704	-4.0711709
H	-2.3276835	-1.0302136	-4.3946707
H	-1.0336213	-3.7179672	2.8571254
H	-2.0094006	0.0955646	4.5736198
H	-1.5490045	-2.3171390	4.8222897
C	-2.3001572	1.9364283	1.0109498
C	-2.9682718	3.1546828	0.8838703
C	-3.3237891	3.6014334	-0.3792977
C	-3.0562099	2.8526103	-1.5147241
C	-2.3876446	1.6349595	-1.3835984
H	-3.2091368	3.7351609	1.7648138
H	-3.8403526	4.5467600	-0.4793723
H	-3.3640005	3.1992886	-2.4925361
N	-2.1129522	0.8365935	-2.4881291
N	-1.9403505	1.4332685	2.2557890
N	-0.8904824	-2.8190303	0.3395901
H	-0.7586756	-3.8087064	0.4598547
H	-2.2768692	1.9408163	3.0566112
H	-2.5118600	1.1332272	-3.3628401
C	5.2248477	0.2524714	0.3025426
C	3.9342807	-0.4616393	-0.0379931
C	2.8957731	0.6715019	-0.2368182
C	3.6884847	1.9334865	0.0194105
C	3.7715013	1.2663363	-1.3237662
C	4.7973406	0.1400815	-1.1295429
C	4.9144361	1.6269682	0.8459516
H	3.1346661	2.8545877	0.1149901
H	4.6984026	1.6082974	1.9136120
H	5.7259757	2.3295061	0.6541510
C	3.6513980	-1.8297003	0.2286013
N	3.4231702	-2.9358321	0.4371242
C	1.4871371	0.5111329	-0.1655574
H	6.0527536	-0.3436335	0.6547414
H	5.3362125	-0.3924080	-1.8947861
H	3.4516521	1.7002478	-2.2557280

2a Norbornadiene-me-phenyl-ethinyl-TATA

$E_{M062x\text{-}D3/def2TZVPP}$ = -1605.175260624

Nimag = 2 (-14.49 cm^{-1}; -5.84 cm^{-1})

C	-0.2457023	-1.4876625	0.6394580
C	-0.5345119	-2.8676838	1.0900366
C	0.7645750	-3.6248130	1.2443716
C	-1.2622650	-2.8201868	2.4137094
C	-1.4012621	-3.5439317	0.0519411
C	-0.8075457	-4.2041110	-1.0202745
C	-1.5980179	-4.6969740	-2.0579207
C	-2.9730723	-4.5329856	-1.9949587
C	-3.5806782	-3.9147659	-0.9130747
C	-2.7862046	-3.4233285	0.1223341
C	-0.5378466	-2.7990450	3.6024283
C	-2.6503133	-2.7136067	2.4365560
C	-3.3135472	-2.5177101	3.6480923
C	-2.5748045	-2.4519134	4.8192594
C	-1.1970648	-2.6040793	4.8162472
H	-4.3912342	-2.4230832	3.6670754
H	-3.0871473	-2.2969734	5.7594210
H	-0.6348968	-2.5765825	5.7403275
H	-4.6567694	-3.8121724	-0.8663231
H	-1.1383084	-5.2010861	-2.8979447
H	-3.5872371	-4.9109227	-2.8011110
C	1.3142258	-4.2850416	0.1485604
C	2.5813256	-4.8598954	0.2459864
C	3.2679201	-4.7802511	1.4476407

C	2.7135300	-4.1658290	2.5597123
C	1.4471049	-3.5902321	2.4571918
H	3.0149114	-5.3643891	-0.6075029
H	4.2514598	-5.2240593	1.5249284
H	3.2498093	-4.1324687	3.4988770
N	0.8404326	-2.9726842	3.5454905
N	0.5756259	-4.3506219	-1.0278267
N	-3.3446155	-2.8032974	1.2353062
H	-4.3483933	-2.8206550	1.2995923
H	0.9607517	-4.9145682	-1.7666791
H	1.3179713	-3.0511334	4.4274931
C	0.9680124	6.9793596	-2.7339705
C	0.9649280	5.4453064	-2.8762942
C	0.8816211	4.9176407	-1.6438303
C	0.8239345	6.1198949	-0.6802712
C	-0.4940389	6.8555527	-0.9575329
C	-0.4094463	7.3661454	-2.1793393
C	1.7903035	7.0708631	-1.4275572
H	1.0396913	5.8789458	0.3557012
H	2.7982860	6.6717841	-1.5273003
H	1.8036216	8.0725182	-1.0031535
C	0.8718669	4.7838203	-4.1256252
N	0.7937828	4.2809286	-5.1570715
C	-0.0232352	-0.3705807	0.2531119
H	1.3146740	7.5238232	-3.6054905
C	1.4605920	2.4533002	-1.6987746
C	0.7241607	3.5368517	-1.1888772
C	-0.2146732	3.3100529	-0.1731754
C	0.2309185	0.9542328	-0.2180916
C	-0.4770424	2.0426037	0.3000452
C	1.1963954	1.1829498	-1.1961089
H	1.7625446	0.3398852	-1.5707817
C	2.5472956	2.6035233	-2.7274277
H	-0.7652839	4.1514835	0.2292815
H	-1.2197137	1.8793297	1.0678276
H	2.1558548	2.4738581	-3.7366770
H	3.3158407	1.8496040	-2.5663322
H	3.0121400	3.5871838	-2.6845659
H	-1.1656789	7.8992154	-2.7341704
H	-1.3355569	6.8786681	-0.2819695

2b Quadricyclane-ethinyl-TATA

$E_{M062x-D3/def2TZVPP}$ = -1605.149206215

Nimag = 2 (-20.93 cm^{-1}; -9.38 cm $^{-1}$)

C	-0.4920794	-1.4529178	0.5637063
C	-0.6497178	-2.8474594	1.0339735
C	0.6905283	-3.5411787	0.9486421
C	-1.1341775	-2.8431952	2.4661788
C	-1.6521433	-3.5606216	0.1557829
C	-1.2252173	-4.1886762	-1.0113497
C	-2.1615047	-4.7197010	-1.8983923
C	-3.5100246	-4.6290828	-1.5908788
C	-3.9465520	-4.0463391	-0.4111764
C	-3.0068563	-3.5154283	0.4722432
C	-0.2122939	-2.7884330	3.5081096
C	-2.4994852	-2.8126794	2.7361005
C	-2.9466326	-2.6632881	4.0488097
C	-2.0164256	-2.5644119	5.0717509
C	-0.6548805	-2.6381868	4.8222882
H	-4.0073038	-2.6289898	4.2599392
H	-2.3616269	-2.4451470	6.0899393
H	0.0604890	-2.5835176	5.6323330
H	-5.0008288	-4.0033645	-0.1714096
H	-1.8328189	-5.1984179	-2.8113169
H	-4.2372912	-5.0393975	-2.2786082
C	1.0710642	-4.1663107	-0.2357381
C	2.3648607	-4.6692074	-0.3724295
C	3.2503876	-4.5512686	0.6874022
C	2.8711554	-3.9696366	1.8874712
C	1.5764238	-3.4681948	2.0196116
H	2.6662243	-5.1455667	-1.2959930
H	4.2552340	-4.9375438	0.5814181
H	3.5650125	-3.9040339	2.7151366
N	1.1413604	-2.8839265	3.2047149
N	0.1395976	-4.2686432	-1.2623338
N	-3.3908861	-2.9311621	1.6751103
H	-4.3640119	-3.0099840	1.9180068
H	0.4174890	-4.7994281	-2.0704374
H	1.7711986	-2.9396377	3.9871547
C	1.7019674	6.8108616	-2.8178765
C	1.2703154	5.3728702	-3.0219623
C	0.5161198	4.9838844	-1.7199147
C	0.6297525	6.2324134	-0.8755158
C	-0.4356858	6.1274493	-1.9385272
C	0.2938146	6.5132441	-3.2347611
C	1.7652195	7.1124688	-1.3402804
H	0.3357150	6.1603382	0.1610384
H	2.7217615	6.8264237	-0.9024730
H	1.5803935	8.1676805	-1.1365444
C	1.8153984	4.4849070	-3.9889471
N	2.2584441	3.7623320	-4.7646901
C	-0.3406944	-0.3260744	0.1716934
H	2.3854789	7.2403303	-3.5344409
H	-0.1472328	6.8140257	-4.1701537
H	-1.4960300	6.1783506	-1.7529481
C	1.3828491	2.8335390	-0.8097147
C	0.2912287	3.6098636	-1.2288832
C	-0.9923770	3.0768596	-1.1789070
C	-0.1392530	1.0086375	-0.3004507
C	-1.2157592	1.7910799	-0.7147000
C	1.1510065	1.5448698	-0.3551056
H	1.9824647	0.9340598	-0.0279695
C	2.7767330	3.3963533	-0.8425132
H	-1.8260071	3.6794573	-1.5156086
H	-2.2159864	1.3829082	-0.6769466
H	3.1174578	3.5506670	-1.8680076
H	3.4785479	2.7231449	-0.3551211
H	2.8160902	4.3620323	-0.3355287

3a Norbornadiene-TOTA

$E_{M062x-D3/def2TZVPP}$ = -1318.251824257

Nimag = 0

C	0.0303869	-0.3511217	0.0093937
C	1.3420349	-1.0221292	0.2740226
C	-0.7457136	-0.3070325	1.2894456
C	-0.7297440	-1.1668789	-0.9776950
C	-0.0526605	-1.8771252	-1.9544285
C	-0.7483791	-2.5153255	-2.9666958
C	-2.1361776	-2.4470080	-2.9580989
C	-2.8358062	-1.7987171	-1.9477594
C	-2.1144738	-1.1678017	-0.9497075
C	-0.0882837	-0.2360257	2.5078261
C	-2.1323889	-0.3281868	1.2480166
C	-2.8704806	-0.1641679	2.4103500
C	-2.1904889	-0.0186140	3.6133187
C	-0.8033025	-0.0741643	3.6843094
H	-3.9495247	-0.1672460	2.3630357
H	-2.7577960	0.1083719	4.5251978
H	-0.2781967	-0.0107475	4.6261669
H	-3.9151937	-1.7686390	-1.9316991
H	-0.2096073	-3.0428432	-3.7398128
H	-2.6892449	-2.9282005	-3.7525818
C	1.9566344	-1.7459952	-0.7380835
C	3.2168738	-2.2907561	-0.5446882
C	3.8271663	-2.1296525	0.6931762
C	3.1928158	-1.4785037	1.7444906
C	1.9342613	-0.9413044	1.5248404
H	3.6902777	-2.8390446	-1.3460414
H	4.8098448	-2.5512750	0.8532533
H	3.6472956	-1.3961991	2.7209928
C	0.2441183	3.1763812	-1.4434643
C	-0.1432466	1.6879500	-1.5909415
C	0.2480325	1.0660811	-0.4730302
C	0.9069482	2.1357278	0.4133587
C	-0.2121972	3.1072510	0.8141606
C	-0.6039523	3.7295868	-0.2906174
C	1.5817329	2.9810505	-0.6931381
H	1.5267044	1.7560412	1.2190608
H	2.3195066	2.4244061	-1.2685206
H	1.9969285	3.9128835	-0.3156694
C	-0.8639888	1.2073038	-2.7175994
N	-1.4372901	0.9026812	-3.6666912
H	0.2450549	3.7415778	-2.3689055
H	-1.4054541	4.4419521	-0.4081380
H	-0.6177832	3.1861946	1.8113661
O	1.2824772	-0.3324287	2.5730844
O	1.3227328	-1.9451889	-1.9370433
O	-2.7904529	-0.5231175	0.0621837

3b Quadricyclane-TOTA

$E_{M062x-D3/def2TZVPP}$ = -1318.226393475

Nimag = 0

C	0.0061829	-0.2663180	0.1019546
C	1.2952103	-0.9497219	0.4240347
C	-0.8673625	-0.3232148	1.3141530
C	-0.6628669	-1.0177742	-1.0034119
C	0.1051800	-1.6523562	-1.9689094
C	-0.4891891	-2.2507742	-3.0667525
C	-1.8772693	-2.2367703	-3.1552149
C	-2.6681507	-1.6908088	-2.1538345
C	-2.0459605	-1.1015356	-1.0619001
C	-0.2948222	-0.2693854	2.5765276
C	-2.2430847	-0.4371384	1.1872189
C	-3.0602172	-0.3620850	2.3051467
C	-2.4677453	-0.2226779	3.5521713
C	-1.0856375	-0.2002219	3.7100225
H	-4.1321166	-0.4116404	2.1839295
H	-3.0974024	-0.1590752	4.4287937
H	-0.6238301	-0.1446450	4.6850072
H	-3.7468299	-1.7257810	-2.1963387
H	0.1249975	-2.7274976	-3.8165603
H	-2.3541478	-2.6964228	-4.0101575
C	1.9987600	-1.6096470	-0.5709343
C	3.2481389	-2.1504571	-0.3092725
C	3.7637243	-2.0336771	0.9764523
C	3.0509072	-1.4226615	2.0010432
C	1.8019465	-0.8927239	1.7130851
H	3.7898972	-2.6541903	-1.0965201
H	4.7372488	-2.4523368	1.1912893
H	3.4391516	-1.3622045	3.0073400
O	1.0759732	-0.3068098	2.7199027
O	1.4751725	-1.7192327	-1.8361984
O	-2.8247284	-0.6292435	-0.0395596
C	-0.0171503	3.3606456	-1.3172645
C	-0.7139158	2.3431798	-0.4315860

C	0.2990561	1.1706492	-0.3030867
C	1.4564655	1.6131371	-1.1602513
C	1.3228780	2.1242525	0.2541833
C	1.0279293	2.6658945	-2.1535188
H	2.2380206	0.9017713	-1.3801845
C	-2.1144788	2.3768862	-0.1845161
N	-3.2436121	2.4471762	0.0161454
C	0.3298704	3.2887176	0.1368149
H	-0.6051590	4.1896293	-1.6807240
H	0.6067968	2.2307517	-3.0597319
H	1.8444056	3.3368115	-2.4216740
H	0.1556229	4.0733537	0.8535434
H	2.0421408	1.9561725	1.0393432

References

[1] Gunes, Y.; Arcelik, N.; Sahin, E.; Fleming, F. F.; Altundas, R. *Eur. J. Org. Chem.* **2015**, 6679-6686.

[2] Laursen, B. W.; Krebs, F. C. *Chem. Eur. J.* **2001**, *7*, 1773-1783.

[3] Browne, D. L.; Baumann, M.; Harji, B. H.; Baxendale, I. R.; Ley, S. V. *Org. Lett.* **2011**, *13*, 3312-3315.

[4] Martin, J. C.; Smith, R. G. *J. Am. Chem. Soc.* **1964**, *11*, 2252-2256.

[5] Kenndoff, J.; Polborn, K.; Szeimies, G. *J. Am. Chem. Soc.* **1990**, *112*, 6117-6118.

[6] Tranmer, G. K.; Yip, C.; Handerson, S.; Jordan, R. W.; Tam, W. *Can. J. Chem.* **2000**, *78*, 527-535.

[7] Gunes, Y.; Arcelik, N.; Sahin, E.; Fleming, F. F.; Altundas, R. *Eur. J. Org. Chem.* 2015, 6679-6686.

[8] Zhao, Y.; Truhlar, D. G., *Theor. Chem. Account* **2008**, *120*, 215-241.

[9] Grimme, S.; Antony, J.; Ehrlich, S.; Krieg, H., *J. Chem. Phys.* **2010**, *132*, 154104.

[10] Weigend, F.; Häser, M.; Patzelt, H.; Ahlrichs, R., *Chem. Phys. Lett.* **1998**, *294*, 143.

[11] Goerigk, L.; Hansen, A.; Bauer, C.; Ehrlich, S.; Najibi, A.; Grimme, S., *Phys. Chem. Chem. Phys.* **2017**, *19*, 32184-32215.

[12] Turbomole7.2: TURBOMOLE V7.2 2017, a development of University of Karlsruhe and Forschungszentrum Karlsruhe GmbH, 1989-2007, TURBOMOLE GmbH, since 2007; available from http://www.turbomole.com.
